# Genome-Wide Association Study of Salinity Tolerance During Germination in Barley (*Hordeum vulgare* L.)

**DOI:** 10.3389/fpls.2020.00118

**Published:** 2020-02-21

**Authors:** Edward Mwando, Yong Han, Tefera Tolera Angessa, Gaofeng Zhou, Camilla Beate Hill, Xiao-Qi Zhang, Chengdao Li

**Affiliations:** ^1^ Western Barley Genetics Alliance, College of Science, Health, Engineering and Education, Murdoch University, Perth, WA, Australia; ^2^ Western Australian State Agricultural Biotechnology Centre, Murdoch University, Perth, WA, Australia; ^3^ Department of Primary Industries and Regional Development Government of Western Australia, Perth, WA, Australia

**Keywords:** genome-wide association, barley, quantitative trait nucleotide, germination, salinity tolerance, genetic marker, marker-trait association

## Abstract

Barley seeds need to be able to germinate and establish seedlings in saline soils in Mediterranean-type climates. Despite being a major cereal crop, barley has few reported quantitative trait loci (QTL) and candidate genes underlying salt tolerance at the germination stage. Breeding programs targeting salinity tolerance at germination require an understanding of genetic loci and alleles in the current germplasm. In this study, we investigated seed-germination-related traits under control and salt stress conditions in 350 diverse barley accessions. A genome-wide association study, using ~24,000 genetic markers, was undertaken to detect marker-trait associations (MTA) and the underlying candidate genes for salinity tolerance during germination. We detected 19 loci containing 52 significant salt-tolerance-associated markers across all chromosomes, and 4 genes belonging to 4 family functions underlying the predicted MTAs. Our results provide new genetic resources and information to improve salt tolerance at germination in future barley varieties *via* genomic and marker-assisted selection and to open up avenues for further functional characterization of the identified candidate genes.

## Introduction

Soil salinity is a major global environmental factor limiting plant growth and productivity (Allakhverdiev et al., 2000; Ashraf et al., 2015). It causes two types of stress in plants, namely osmotic pressure associated with non-ionic factors, and ionic stress induced by Na^+^ and Cl^−^ ions (Bernstein, 1975; reviewed by Munns and Tester, 2008). High salt in the soil increases the osmotic pressure and creates a condition similar to drought (Leon, 1963; Bliss et al., 1986; Sayar et al., 2010) that impairs the ability of seeds to absorb water from the soil, hence prolonging or even inhibiting seed imbibition for subsequent germination. In addition, the absorption of excess Na^+^ and Cl^−^ ions causes toxicity that impedes normal cellular processes (Hampson and Simpson, 1990), contributing to a decrease in seed germination rate (Dodd and Donovan, 1999; Zhihui et al., 2014). Ionic and osmotic stress interaction effects ultimately decrease the number of sprouted seeds and the germination rate (Kazemi and Eskandari, 2011).

Barley is one of the most saline-tolerant crops (Munns, 2005) and is often used as a model to understand salinity adaptation mechanisms in plants (Chen et al., 2007; Wu et al., 2011). Adaptation to salinity varies among barley genotypes and growth stages (Mano et al., 1996; Mano and Takeda, 1997; Xue et al., 2009). The germination process begins when a quiescent dry seed imbibes water, and terminates on the emergence of the radicle (Gupta et al., 2019); barley is a model plant for studying the germination stage in monocots (Gorzolka et al., 2016). Depending on their ability to germinate and survive under salinity stress, barley genotypes are either tolerant or sensitive depending on their genetic diversity (Shelden et al., 2013; Shelden et al., 2016; Gupta et al., 2019). At this stage, several different loci control salinity tolerance (Mano and Takeda, 1997). Angessa et al. (2017) reported transgressive phenotypic segregation for germination rate and biomass at the seedling stage using a doubled haploid (DH) barley population derived from a CM72 Gairdner cross, with both traits controlled by different QTLs on chromosomes 2H and 3H, respectively. At the germination stage, Mano and Takeda (1997) reported 17 QTLs controlling abscisic acid (ABA) response on chromosomes 2H, 3H, 1H, and 5H in Steptoe Morex DH lines, and 9 QTL on 2H and 5H in a Harrington TR306 DH population. Loci located on chromosome 5H in both populations were closely linked to salinity tolerance. QTL mapping using a DOM REC Oregon Wolf Barley population identified several chromosomal regions on 2H, 5H, and 7H that were associated with salt stress response at the germination stage (Witzel et al., 2009). A single QTL on chromosome 5H, detected at three salt concentrations, was responsible for 42% of the phenotypic variation (Cattivelli et al., 2002).

There is little information linking the QTLs reported for salinity tolerance at the germination stage to specific genes and genetic mechanisms (Mano and Takeda, 1997; Hanen et al., 2014; Angessa et al., 2017). Genome-wide association (GWAS) studies are increasingly used to discover and explain the genetic basis of agronomic traits that are often controlled by many genes of small magnitude, such as germination (Shi et al., 2017; Hazzouri et al., 2018; Naveed et al., 2018; Yu et al., 2018). GWAS relies on linkage disequilibrium (LD) to detect associations between a large number of genetic variants and traits across a large number of genotypes from natural populations. GWAS typically achieves higher mapping resolution due to higher recombination levels between the linked genetic loci and traits at the population level than conventional QTL mapping (Hu et al., 2011). With the current advances in genome-wide genotyping technology, hundreds of accessions encompassing thousands of gene loci can be genotyped using high-throughput markers to improve the efficiency of current breeding approaches (Russell et al., 2011; Kilian and Graner, 2012; Tondelli et al., 2013). GWAS can precisely locate polymorphisms and the underlying genetic loci that are accountable for phenotypic variations to allow gene-targeted searches (Naveed et al., 2018; Xu X. et al., 2018; Yu et al., 2018).

This study used GWAS analysis to identify salinity tolerance at the seed germination stage in 350 barley accessions from 32 countries. The germination rate of these accessions was assessed in 150 mM NaCl, and a tolerance index was calculated (the fraction of germination under salt and deionized water as a percentage), using seeds harvested from two trial locations in Western Australia. The GWAS analysis of two traits associated with salinity tolerance at germination was conducted using 24,138 diversity arrays technology (DArTseq) and single-nucleotide polymorphism (SNP) markers. This research aimed to identify quantitative trait nucleotides (QTNs) and predict genes that are highly associated with salt-tolerant traits at the germination stage to select markers for future breeding.

## Materials and Methods

### Barley Germplasm

A total of 350 barley genotypes selected from a larger set of 594 accessions in a worldwide collection were evaluated for salinity response at the germination stage to map the locations of genes associated with tolerance (**Supplementary Tables 1** and **2**). The genotypes originated from 32 countries in various geographic regions, including Europe, Asia, North and South America, Africa, and Australia (**Supplementary Figure 1**), and comprised landraces, domesticated cultivars, and breeding lines conserved at the Western Barley Genetics Alliance at Murdoch University Perth, Australia. The domesticated barleys were selected from various breeding programs representing all cultivated varieties, including two-row (92%) and six-row (8%) head types, and winter (7%), spring (92%), and facultative (1%) growth habits (**Supplementary Table 2**). All barley plants were grown at two Western Australian locations, Merredin (31.4756°S, 118.2789°E, 315 m asl, 324 mm annual rainfall) and Katanning (33.6856°S, 117.6064°E, 320 m asl, 470 mm annual rainfall) in the 2016 and 2017 cropping seasons, respectively, and harvested at maturity. Both sites experience Mediterranean-type climates with hot, dry summers, and winter-dominant rainfall (**Supplementary Figure 2**) and are affected by salinity. The hot, dry summer increases salinity levels through ion accumulation in the topsoil, just before the autumn sowing, that affects seed germination. After harvest, the seeds were stored for at least 2 months at room temperature and then incubated at 37°C for 48 h to break seed dormancy.

### Evaluation of Salinity Tolerance at Germination

This study used a modified method based on those of Bliss et al. (1986) and Angessa et al. (2017) to determine salinity tolerance during germination. Barley seeds were surface-sterilized for 5 min using 10% sodium hypochlorite, and then rinsed with sterile water. A set of 100 seeds of each genotype was placed in a 90 mm Petri dish on two layers of Whatman no. 1 filter paper to germinate. The treatments, with three replicates per treatment, contained either 4 ml deionized (DI) water (control) or 150 mM NaCl (salt treatment). The Petri dishes were sealed with parafilm and placed in a dark oven at 20°C. Germinated seeds were counted after 72 h of incubation; most domesticated barley malt varieties (mostly two-row)—selected for dormancy are expected to germinate (95–100%) within 2–4 days of imbibition (Briggs, 1978; Bothmer et al., 1995). However, the wild form (ssp. *Spontaneum*), those developed for feed, and most six-row varieties have not undergone such selection; hence, seed germination is irregular (Oberthur et al., 1995). To account for this variation, the tolerance index (TI) was adopted to reflect the stress effect on the same genotype over the period; any reduction in germination was considered to be caused by salinity stress (Askari et al., 2016). The germination percentage (G%) was calculated following the equation of Adjel et al. (2013), namely, G% = GS/TS×100%, where GS is the total number of germinated seeds, and TS is the total number of incubated seeds. The tolerance index was subsequently calculated as follows (Angessa et al., 2017), TI% = G_t_%/G_c_%×100%, where G_t_% is the percentage of seeds germinated in the salt treatment, and G_c_% is the percentage of seeds germinated in deionized water.

### Statistical Analysis

The germination percentage of individual accessions from three replications and two locations were analyzed by SAS software (version 9.4, SAS Institute Inc, 2013). Analysis of variance (ANOVA) was performed to test the interaction between germplasm, treatments, and locations. Correlation analysis between germination in the salt treatment and the tolerance index was calculated and visualized using IBM SPSS Statistics (Version 25.0, IBM Corp, 2017).

### Genome-Wide Marker Profiling

We used a combination of three sequencing methods to capture variation in and around the gene-containing regions of 350 barley genotypes, namely targeted resequencing, low-coverage whole-genome resequencing, and DArTseq. We used SNP markers, based on a custom target-enrichment sequencing assay, that included loci implicated in the flowering pathway in barley and related plant species, as previously published by Hill et al. (2019a, 2019b). The remainder of the pre-capture DNA libraries were subjected to low-coverage whole-genome sequencing at BGI (Hongkong) on an Illumina HiSeq4000 instrument. DArTseq genotyping by sequencing (GBS) was performed using the DArTseq platform (DArT PL, Canberra, NSW, Australia) as described on the company website (https://www.diversityarrays.com/). The genetic position of each marker was determined based on the Morex physical reference assembly. All sequence files were post-run filtered and aligned to barley reference genome assembly (IBSC v2; Mascher et al., 2017). All genotype data were combined, filtered for duplicates, minor allele frequency (MAF), and imputed using BEAGLE v4.1 adopting MAF > 0.05, SNP call rate > 0.95, and missing values < 0.05 (Browning and Browning, 2007).

### Population Structure and Linkage Disequilibrium Analysis

Population structure was analyzed by Structure software version 2.3 (Hubisz et al., 2009). The genotypic data were imported into the software; the burn-in period was set to 5,000, producing 5,000 MCMC (Markov Chain Monte Carlo) repetitions. Simulations were conducted to estimate the number of populations (K) using admixture models by running K from 2 to 10, as described by Evanno et al. (2005). The LD between every two linear markers and the correlation between a pair of markers, which is squared allele frequency correlations (*r* ^2^ value), was estimated using TASSEL software version 5.0 (Bradbury et al., 2007). Correlations between a pair of markers (*r* ^2^ value) and the genetic distance was selected to calculate LD using a fitted equation in the whole genome.

### Genome-Wide Association Analysis

Marker-trait association analysis for the salinity tolerance index at germination was performed by TASSEL software version 5.0 (Bradbury et al., 2007), using the mixed linear model basing on: trait of interest = population structure + marker effect + individual + residual. Heritability was estimated with the formula proposed by Kruijer et al. (2015), using genetic variance simulated from tolerance index and marker data obtained from the TASSEL package. The effectiveness and appropriateness of the model were assessed by constructing quantile–quantile (Q–Q) plots. Manhattan plots were constructed to visualize the GWAS output, with chromosome position as the X-axis and –log (*P*-value) of all markers using the R “qqman” (Wickham, 2009; R Core Team, 2014; Bates et al., 2015). Markers with *P* < 0.05 were considered significant and corrected for multiple tests by calculating *q*-value (FDR adjusted *P*-value). False discovery rate (FDR) correction was performed using Benjamini-Hochberg multiple test correction to determine significant marker-trait associations (MTA) (Benjamini and Hochberg, 1995) and markers with *q*-values < 0.05 were selected.

The formula described by Li et al. (2016) was used to identify favorable alleles for markers significantly associated with salinity tolerance during germination. The tolerance allele effect (ai) was calculated as; a_i_ = ∑x_ij_/n_i_ − ∑N_k_/n_k_, where a_i_ is the tolerance effect of the i_th_ allele, x_ij_ is the tolerance index value over the j_th_ material with the i_th_ allele, n_i_ is the number of germplasm with the i_th_ allele, N_k_ is the salinity tolerance value across all genotypes, and n_k_ is the number of germplasm (Mei et al., 2013). In this study, *ai* denotes the association of the average salinity tolerance value of germplasm with a specific allele with that of all genotypes; hypothetically, values > 0 have a positive effect on the trait, and < 0 have a negative effect (Zhang et al., 2013).

### Database Search to Predict Putative Candidate Genes and Favorable Alleles

The barley reference genome assembly (IBSC v2; Mascher et al., 2017) was used to identify possible candidate genes by searching the region flanking the QTN range of significantly associated salinity tolerance markers, with a –log10 (P) (logarithm of the odds –LOD) value set at ≥ 3.

## Results

### Phenotypic Variation and Correlations Among Traits

Three hundred and fifty barley genotypes were evaluated for salinity tolerance under control (germination in DI water) and salt conditions (150 mM NaCl) using seeds obtained from barley grown in Merredin and Katanning (Western Australia). The ANOVA results showed that genotype, treatment, and location differed significantly at *P* < 0.01. There were significant interactions between genotype and treatment and genotype by treatment by location (**Table 1**). In the salt treatment, the tolerance index and germination percentage had a positive correlation, such that a high percentage for the two traits indicated tolerance to salinity stress. There was a positive correlation coefficient between germination at 150 mM NaCl and tolerance index (*R^2^* = 0.85 for Merredin and *R^2^* = 0.90 for Katanning; see *Supplementary Information*), indicating that either of the two can be used to identify salinity tolerance during germination (**Supplementary Figure 3**).

**Table 1:** Phenotype analysis of variance (ANOVA) for barley germplasms.

Source	DF	SS	Mean square	F value
Germplasm	349	156,424.66	448.2081948	17.99*
Treatment	1	298,428.89	298,428.89	18,018.50*
Location	1	1,563.57	1,563.57	94.41*
Germplasm × treatment	349	76,629.44	219.568596	8.81*
Germplasm × location	349	50,714.36	145.3133524	5.83*
Germplasm × treatment × location	350	17,711.39	50.60397143	2.03*
Error	1,048	26,126.64	24.93	
**Total**	1,412	627,598.95		

*Significant at 1% probability level.

**Figure 1:**
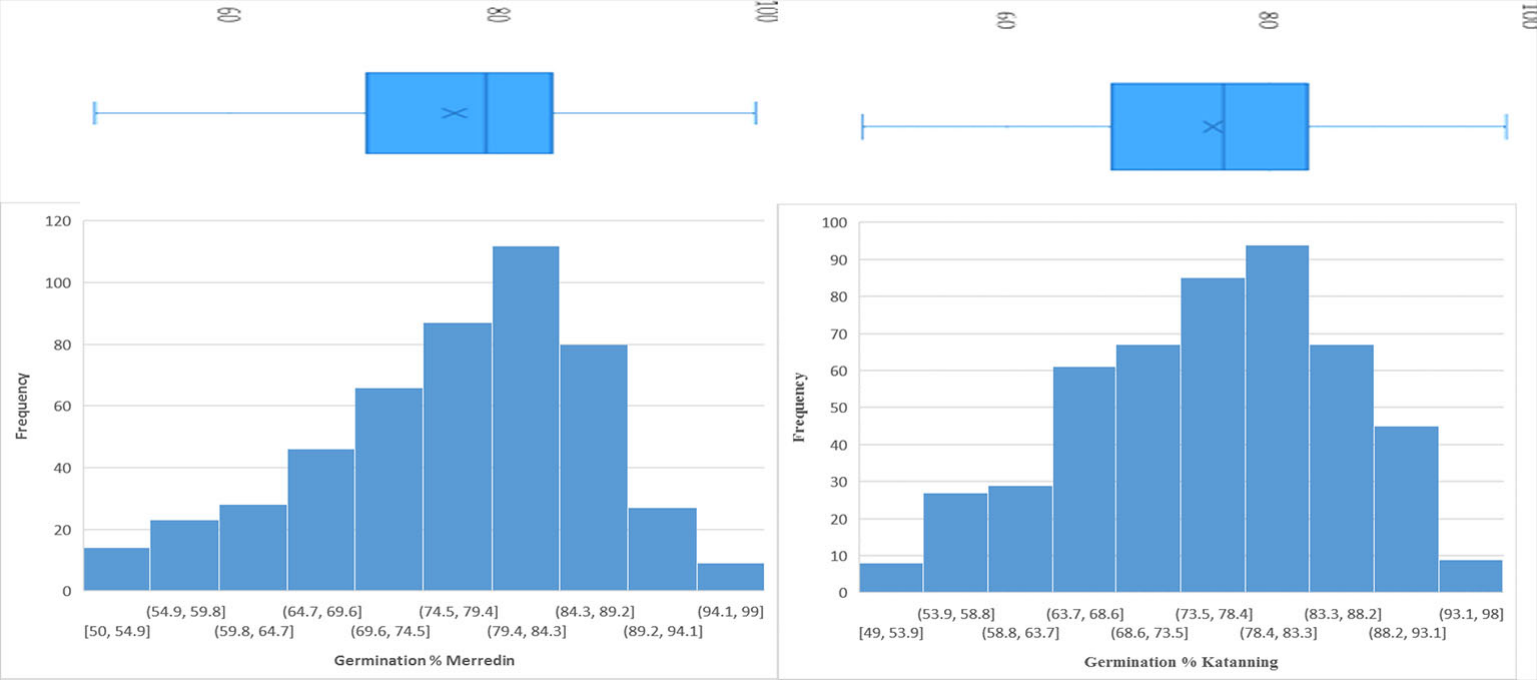
Combined histogram and plot block for germination percentage of 350 barley genotypes under 150 mM NaCl for seeds sourced from Merredin and Katanning, WA.

**Figure 2:**
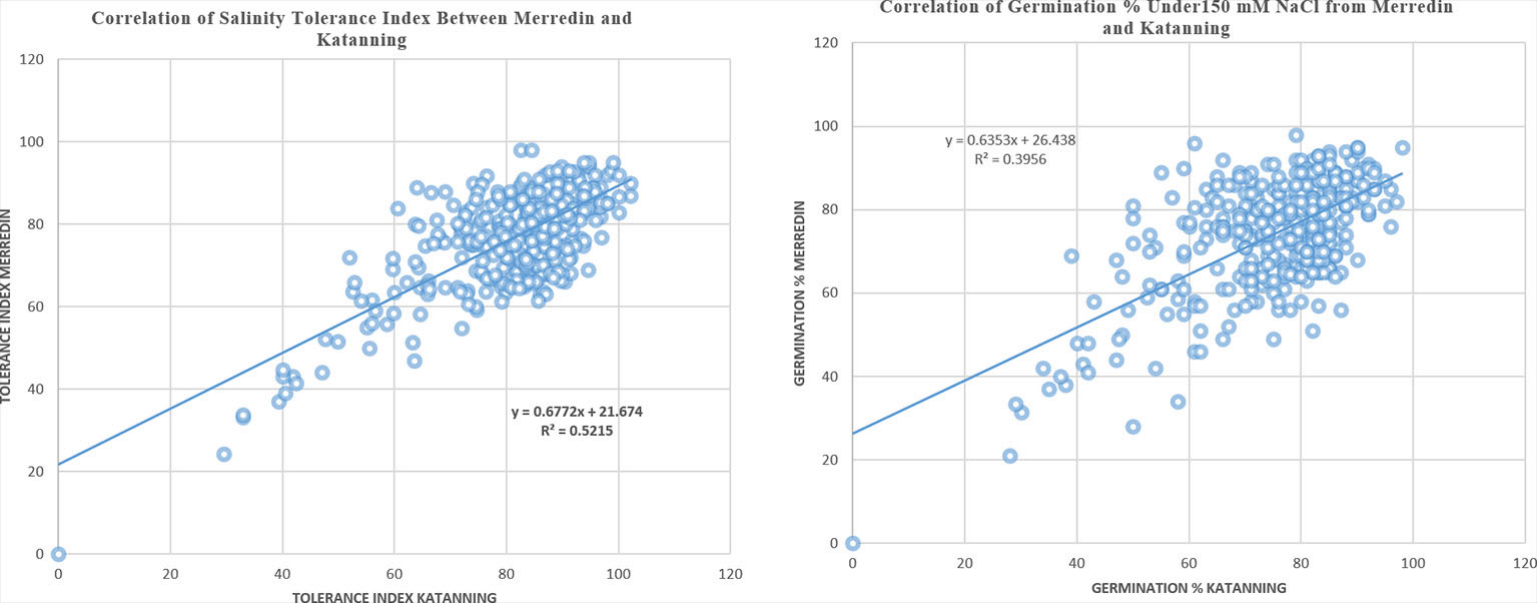
Correlation coefficient for seeds sourced from Merredin and Katanning for tolerance index and germination in 150 mM NaCl.

In the control (DI water), the average germination percentage at the two locations was 94.5% (**Supplementary Figure 5**). In the salt treatment (150 mM NaCl), the average germination percentage at Merredin was 76.8% (range 50–99%) and Katanning was 75.7% (range 49–98%) (**Figure 1**). The high average germination percentage in DI water indicates that the seeds were not dormant; therefore, the reduction in germination in the salt treatment can be attributed to salinity stress. The mean tolerance index for the two locations was 79.5%, with an average of 96.99% for the most tolerant germplasm WABAR2347 (**Table 2**) and 52.73% for the susceptible Torrens (**Supplementary Table 4**). At both sites (Merredin and Katanning), the tolerance index and germination rate in 150 mM NaCl had positive correlations of *R^2^* = 0.52 and 0.40, respectively (**Figure 2**). The top 10 genotypes at each location in terms of tolerance index are presented in **Table 2** and **Supplementary Figure 4**.

**Table 2:** List of most tolerant barley accessions (top 30) for seeds sourced from Merredin and Katanning (Western Australia).

S/No.	Accession	Merredin		Katanning		Average	
		Germination %	Tolerance index	Germination %	Tolerance index	Germination %	Tolerance index
1	WABAR2347	98	98.98	95	95	96.5	96.99
2	Har.Nan-35-	93	100	90	90	91.5	96.09
3	BM9647D-66	94.2	98.67	93	93	89	95.84
4	90SM193-34-	92	97.92	91	91.92	91.5	94.92
5	WVA22	90	94.77	95	95	92.5	94.88
6	WABAR2234	95	102.17	87	87	91	94.59
7	CDCGuardian	90	94.77	94	94	92	94.38
8	Yambla	90	93.75	95	95	92.5	94.38
9	90S205-45-4	89	94.44	94	94	89.5	94.22
10	H92036005Z	90	95.79	87	91.89	88.5	93.84
11	WABAR2425	91	100	92	97.42	91.51	93.32
12	ICB104039	90	96.79	89	89	89.5	92.89
13	ACMETCALFE	89	92.68	92	92	90.5	92.34
14	Patty	92	92	90	92.68	91	92.34
15	DH29287	86	95.56	89	89	87.5	92.28
16	HB08306	90	95.74	87	88.75	88.5	92.25
17	Landlord	82	95.45	89	89	85.5	92.23
18	WI4704	83	91.21	93	93	88	92.1
19	Tallon	88	89.75	94	94	91	91.88
20	CM67	92	92.9	79	90.8	85.5	91.85
21	VB0904	88	95.7	88	88	88	91.85
22	B697	85	96.61	86	86.76	85.5	91.69
23	WI4574	93	93.98	89	89	91	91.49
24	VTAdmiral	93	97.96	85	85	89	91.48
25	85SW:576	96	97.96	85	85	90.5	91.48
26	Mackay	88	90.14	90	92.73	86.5	91.44
27	BM9204-17	91	92.83	90	90	90.5	91.42
28	TR07393	97	100	82	82.78	89.5	91.39
29	CORGI	90	96.76	82	85.37	86	91.06
30	Tore*	86	89	93	93	88	91
	**Mean**	**90.37**	**95.48**	**89.5**	**90.67**	**89.58**	**92.93**

*A representation of the calculate actual TI of the accessions ranked as the top 30.

### Marker Coverage, Population Structure, and Linkage Disequilibrium Analysis

Only DArTseq markers with a call rate of > 95% were selected, being 9,637 from a total of 22,241. An additional 28,502 SNP markers were identified by aligning the low-coverage sequences and targeted re-sequencing (Hill et al., 2019a; Hill et al., 2019b) of 350 barley accessions to the “Morex” reference genome and removing those with less than 5% allele frequency. In total, 24,138 DArTseq and SNP markers, anchored to the barley reference genome, were selected for population structure, linkage disequilibrium, and GWAS analysis.

Population structure analysis combined previously selected DArTseq and SNP markers to determine the genetic background of germplasm belonging to a group in a given number of populations (K). The number of genetic clusters (K values) for population structure was analyzed in 350 barley genotypes with STRUCTURE software where parameter (ΔK) was used to determine the number of clusters suitable for association mapping analysis, with the cluster parameter K set from 2 to 10. The appropriate number of clusters was defined as 3, according to the method by Evanno et al. (2005)—when k was 3, ΔK would reach a top value of 21. The outputs were cross-confirmed to determine the optimal K-value, which was authenticated to be 3, according to the valley of the error rates of cross-validation (**Supplementary Figure 4** and **Supplementary Table 6**).

Linkage disequilibrium (LD) decay (r^2^) of individual chromosomes was analyzed and then summed to obtain an average value for the whole genome. The mean LD decay value for the 350 barley accessions was 3.5 Mb (r^2^ = 0.2), with 24,138 were evenly spread and adequate for GWAS.

### Genome-Wide Association Analysis of Salinity Tolerance at Germination

**Figure 3:**
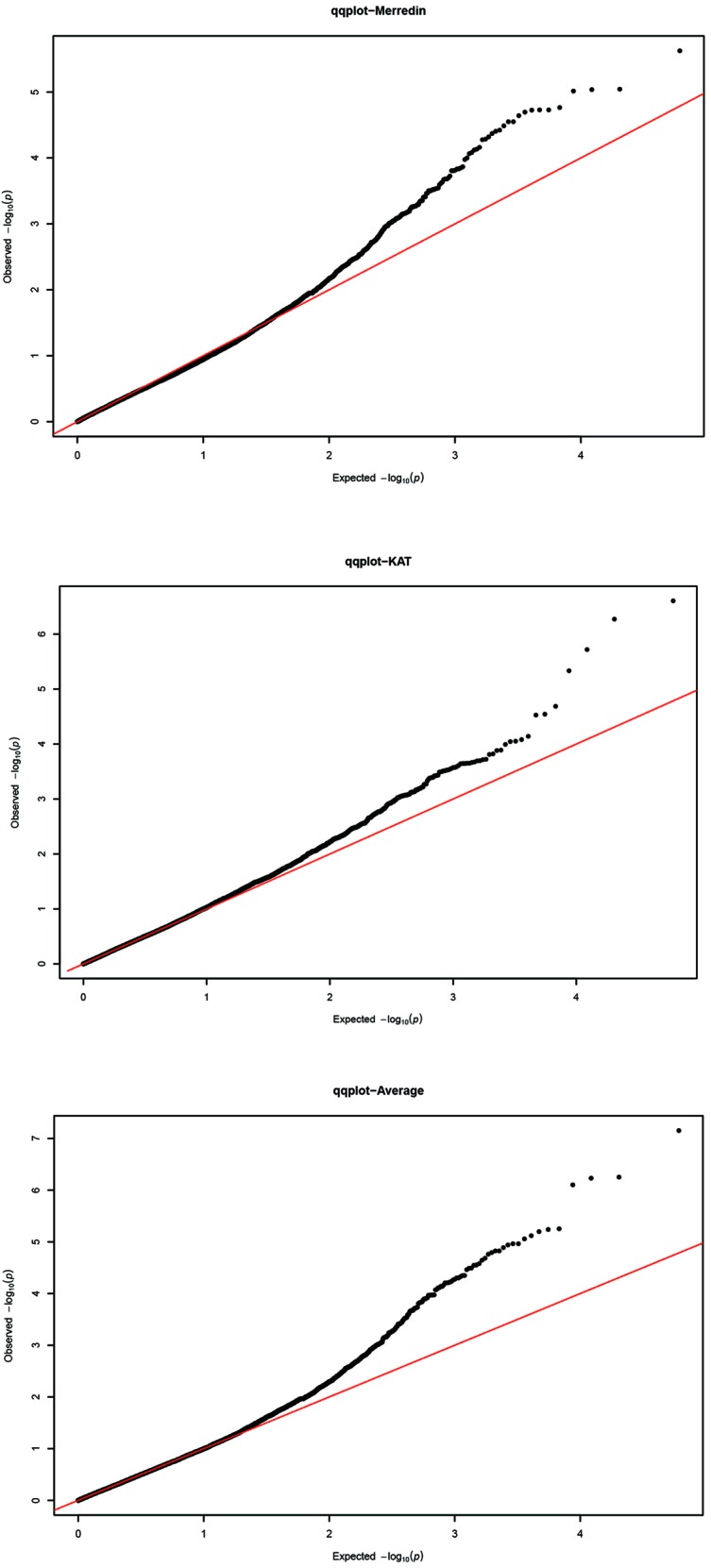
Quantile–quantile (Q-Q) plots for genome-wide association study (GWAS) of 350 barley accessions grown in Merredin, Katanning, and average for salinity tolerance index during germination under 150 mM NaCl. The Y-axis is observed –log (P) values, and X-axis the expected.

**Figure 4:**
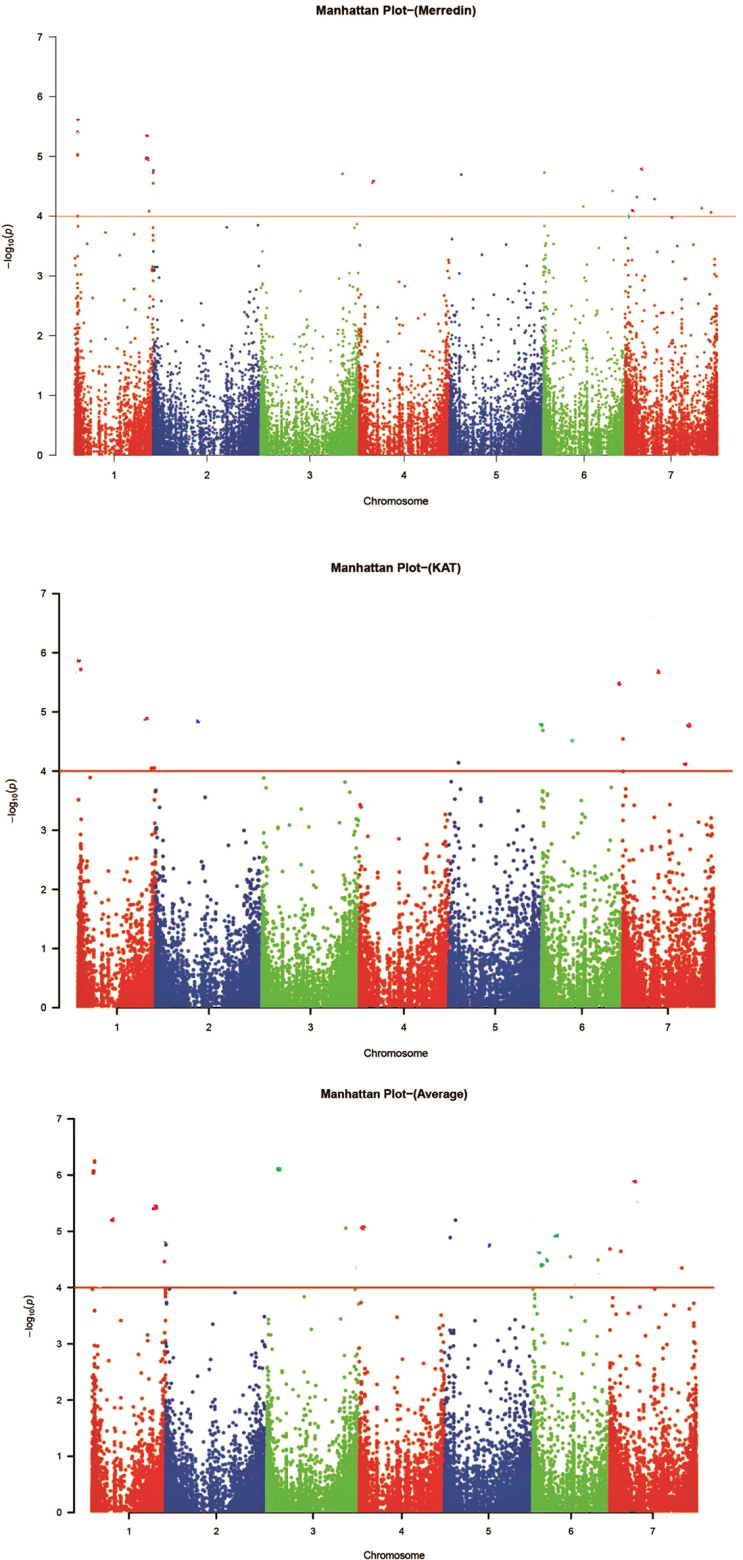
Manhattan plots for genome-wide association study (GWAS) of 350 barley accessions grown in Merredin, Katanning, and average for salinity tolerance index during germination under 150 mM NaCl. Each color indicates a different chromosome, the Y-axis is –log (P) values, and the dots above the red line are significant markers at –log10 (P) ≥ 4.0 (Krzywinski et al., 2009).

The GWAS was performed on 350 genotypes using both genotypic and phenotypic data. Given that the accuracy of association mapping analysis might be affected by population stratification, quantile–quantile (QQ) plots were generated to test the suitability of the model (**Figure 3**). The plots showed that the observed values (ordinate) initially matched the equivalent expected values (abscissa), but eventually, they were delineated and deviated to indicate a reasonable positive. Therefore, the GWAS results from all locations were reliable and not likely to give false negatives due to population stratification. Manhattan figure plots were created to visualize the significance of markers associated with the tolerance index (**Figure 4**). Heritability values of 0.18, 0.11, and 0.19 were obtained from the tolerance indices of Merredin, Katanning, and the average of the two locations, respectively.

### Marker-Trait Associations for Salinity Tolerance Index During Germination

The threshold level was determined at a significance level of *P* = 0.05. Centered on the same, the genome-wide significance threshold for this study was *P* = 1.39×10^−4^ or –log10 (P) = 3.86 (rounded to 4.0). The selected markers were corrected for multiple testing, and those with *q*-values (FDR adjusted *P*-value) < 0.05 were considered accurately significant. Fifty-one markers (18 from Merredin, 13 from Katanning, and 21 from the average of the two sites), associated with the salinity tolerance index at germination were detected across all chromosomes, when –log10 (P) > 4.0. (**Figure 4**). Of these, L1H018492689 explained R^2^ value of 11.03% and D7H016569501 for 6.33% as the highest and lowest, respectively (**Supplementary Table 3**). Five markers were detected at both locations (L1H018492798, L1H018492689, D1H528333687, L7H212035410, and D7H085710245), with eight at Katanning (L2H525371651, L5H070630348, L6H002587116, L6H004005746, D6H074421386, L7H004015622, C7H653619080, and D7H655103370), and 14 at Merredin (L1H018495748, C1H556900705, C1H556900787, D2H001502476, D3H598501321, L4H635824216, L5H044127079, L6H286731484, D6H471369639, L6H495910722, D7H016569501, L7H614807240, and D7H638672485). A hybrid of 21 markers at both locations was detected when the average was used (**Supplementary Table 3**). Some markers were detected only in one location, while others were present in both—an indication that some QTNs showed gene by environment (G×E) interactions.

### Mining of Favorable Marker Alleles Associated With Salinity Tolerance At Germination

The GWAS results are presented in **Figure 3** and **Supplementary Table 3** for significant markers at –log10 (P) > 4.0 and adjusted *P*-value (FDR) < 0.05. All significant markers from the two locations (Merredin and Katanning) were considered, and those detected in a range of 3.5 Mb were pooled to select the marker with the highest –log10 (P). Twelve representative significant markers were selected grounding on –log10 (P) > 4.0, overlapping both locations, and presence in one location and average (**Table 4**). Marker alleles with positive effects for tolerance index were considered favorable alleles, whereas marker alleles with negative effects were deemed unfavorable. Among the favorable marker alleles, L6H495910722, L6H286731484, and L7H614807240 had positive phenotypic effects on salinity tolerance at germination, being 5.3, 1.5, and 1.8%, respectively (**Table 3**). Salinity tolerance at germination in genotypes with favorable marker alleles was greater than those in genotypes with unfavorable marker alleles. Genotypes BM9204-17 and BM9647D-66 had favorable alleles for marker L6H495910722 and were present in the top 10 and 30 list (**Supplementary Table 4** and **Table 2**) of varieties with the highest tolerance index from both locations. Accessions BM9647D-66 and TR06390 had favorable alleles for markers L6H495910722 and L7H614807240, while genotype SM02544 had favorable alleles for markers L6H495910722 and L6H286731484.

**Table 3:** Favourable alleles, their phenotypic effects (ai), and the number of accessions.

Marker	Chromosome	Ref-allele	Alt-allele	Favorable allele	ai * value %	Accessions
L1H018492689	1H	C	T	T	0.377	227
D1H528333687	1H	G	C	G	0.262	299
D2H001502476	2H	G	C	G	0.429	295
L2H525371651	2H	G	A	G	0.291	155
D3H598501321	3H	A	G	G	0.201	299
L5H044127079	5H	G	T	G	0.311	314
L6H286731484	6H	G	A	G	1.521	300
L6H495910722	6H	A	C	C	5.321	21
L7H004015622	6H	C	T	C	0.381	108
L7H212035410	7H	T	C	T	0.475	327
D7H085710245	7H	T	G	T	0.451	331
L7H614807240	7H	C	T	T	1.183	305

### Quantitative Trait Nucleotides Controlling Salinity Tolerance During Germination in Barley

Significant markers flanking a range of 3.5 Mb were considered under one QTN, with only the highest –log10 (P) selected. Nineteen QTNs for salinity tolerance index during germination were identified from the two locations. Two QTLs were mapped on chromosomes 1H, 2H, and 4H, four each on 5H, 6H, and 7H, and one on 3H (**Table 4**). In Katanning, we detected ten QTNs—two on chromosome 1H and 6H, four on 7H, and one on 5H and 2H. Twelve QTNs were detected at the Merredin site, four on 7H, one each on 2H, 3H, 4H, and 5H, and two on 2H and 6H. When the average tolerance index value from the two locations was used for the analysis, a hybrid of the QTNs detected from Katanning and Merredin were realized (**Table 4**). For further analyses, we only considered QTNs that were present at both locations and their average. Four QTNs were present at Merredin, Katanning, and when the average was used, with two each on chromosome 1H and 7H (**Table 5**).

**Table 4:** Association mapping quantitative trait nucleotides (QTNs) for salinity tolerance at germination in barley.

Location	Marker	Chr	Position (bp)	Marker R2	–log10 (P)	q-FDR
Merredin	L1H018492689	H1	18,492,689	0.087	5.514	0.028
	C1H556900705	H1	556,900,705	0.082	5.402	0.028
	D2H001502476	H2	1,502,476	0.087	4.864	0.048
	D3H598501321	H3	598,501,321	0.079	4.869	0.048
	L4H635824216	H4	635,824,216	0.075	4.676	0.050
	L5H044127079	H5	44,127,079	0.082	4.941	0.047
	L6H286731484	H6	286,731,484	0.074	4.261	0.071
	L6H495910722	H6	495,910,722	0.078	4.722	0.049
	D7H016569501	H7	16,569,501	0.063	4.049	0.088
	D7H085710245	H7	85,710,245	0.079	4.420	0.054
	L7H212035410	H7	212,035,410	0.079	4.730	0.049
	L7H614807240	H7	614,807,240	0.072	4.425	0.053
Katanning	L1H018492689	H1	18,492,689	0.102	5.934	0.018
	D1H528333687	H1	528,333,687	0.088	4.915	0.036
	L5H070630348	H5	70,630,348	0.082	4.841	0.037
	L6H004005746	H6	4,005,746	0.087	4.878	0.037
	D6H074421386	H6	74,421,386	0.073	4.482	0.051
	L7H004015622	H7	4,015,622	0.099	5.443	0.020
	D7H085710245	H7	85,710,245	0.071	4.403	0.059
	L7H212035410	H7	212,035,410	0.114	5.816	0.019
	D7H655103370	H7	655,103,370	0.085	5.312	0.029
Average	L1H018492689	H1	18,492,689	0.110	6.352	0.006
	D1H528333687	H1	528,333,687	0.097	5.440	0.013
	D2H001502476	H2	1,502,476	0.090	4.961	0.030
	D3H598501321	H3	598,501,321	0.100	6.357	0.006
	L4H007417825	H4	7,417,825	0.082	5.215	0.016
	L5H017667933	H5	17,667,933	0.090	4.893	0.030
	L5H044127079	H5	44,127,079	0.086	5.289	0.028
	L5H232131131	H5	232,131,131	0.087	4.626	0.035
	L6H015979347	H6	15,979,347	0.075	4.553	0.043
	L6H042597693	H6	42,597,693	0.073	4.396	0.050
	L6H286731484	H6	286,731,484	0.080	4.947	0.023
	L6H495910722	H6	495,910,722	0.079	4.589	0.043
	L7H004015622	H7	4,015,622	0.083	4.984	0.030
	D7H085710245	H7	85,710,245	0.082	4.845	0.030
	L7H212035410	H7	212,035,410	0.100	5.802	0.009
	L7H614807240	H7	614,807,240	0.075	4.496	0.050

**Table 5:** Quantitative trait nucleotides present at both locations, estimated flanking region, and gene numbers.

QTN	Flanking markers	Length of region (bp)	Number of genes
L1H018492689	L1H017315659–L1H018494015	1,178,356	21
C1H556900757	L1H556830379–L1H556830379	1,392,219	30
L7H212035410	L7H212035367–L7H226963761	1,492,840	62
D7H085710245	L7H082317438–D7H085710245	3,392,807	30

The estimated boundaries of the four QTNs were determined using –log10 (P) (logarithm of the odds –LOD) of the markers, setting the threshold at LOD ≥ 3, i.e., the borders for the intervals were the markers immediately below LOD 3. The most significant marker within the borders was selected as the representative QTN in the region. Two QTNs on chromosome 1H were flanked within an average range of 1.18 Mb for marker L1H018492689 and 1.39 Mb for C1H556900757, while on 7H they oscillated at an average of 1.49 Mb for L7H212035410 and 3.39 Mb for D7H085710245.

### Candidate Gene Prediction

A search for possible salt-tolerant candidate genes within the regions flanking each marker, based on the estimated QTNs boundaries above (**Table 5**), was conducted on the recently published barley reference genome assembly, with 143 genes found (**Supplementary Table 5**). Of these, four were very close to the most significant markers, or the markers were inside them; hence, they were given a high confidence as possible candidates (**Table 6**). Genes associated with the following four markers, *Piriformospora indica*-insensitive protein 2 (L1H018492689), lipase 1 (L7H212035410), protein kinase superfamily protein (C1H556900757), and heat shock protein 21 (D7H085710245), most likely play role in enhancing salinity tolerance during germination, as indicated by their –log10 (P) and % R^2^ values (**Supplementary Table 3**). The frequency of B3 domain-containing protein, glutamate-1-semialdehyde-2;1-aminomutase, heat shock protein 21, leucine-rich repeat protein kinase family protein, MADS-box transcription factor family protein, protein kinase superfamily protein, RING/U-box superfamily protein, ubiquitin-like superfamily protein, and zinc finger protein family more than once at different chromosome locations indicated their involvement in enhancing salinity tolerance during germination (**Supplementary Table 5**).

**Table 6:** Genes close to or embedding significant markers associated with salinity tolerance at germination.

Marker	Chromosome	Genes associated ID	Start	End	Function description
L1H018492689	1H	HORVU1Hr1G008420	18,484,404	18,485,253	*Piriformospora indica*-insensitive protein 2
C1H556900757	1H	HORVU1Hr1G094990	556,905,147	556,910,542	Protein kinase superfamily protein
L7H212035410	7H	HORVU7Hr1G053930	212,741,878	212,744,393	Lipase 1
D7H085710245	7H	HORVU7Hr1G036570.2	85,583,651	85,584,754	Heat shock protein 21

## Discussion

### Salt Stress Significantly Inhibited Seed Germination

Seed germination is the first and most crucial stage in crop growth and development (Almansouri et al., 2001). It starts with the imbibition of water, which is repressed in the presence of salinity stress, hence disturbing the progression of germination (Othman et al., 2006). Earlier reports have shown that salinity delays the initiation processes, thus reducing germination percentage and vigor (Dodd and Donovan, 1999; Zhihui et al., 2014). The biochemical and physical processes involved are incredibly complex and attributed to osmotic stress and ionic toxicity (Yu et al., 2018). Barley is a Mediterranean field crop that is directly sown in soil in autumn after hot summer, and salt tolerance during seed germination is essential. In this study, salinity reduced the average germination percentage across locations in the barley germplasm by 18.25%, confirming the compound effect of the stress (El Madidi et al., 2004; Abdi et al., 2016). At both locations, germination under salt stress and the tolerance index had a positive correlation (R^2^ = 0.85–0.90), indicating that adapted barley germplasm has the capacity to withstand salt stress (Munns, 2005; Tajbakhsh et al., 2006; Zhang et al., 2010; Negrão et al., 2017).

Different methods of screening for salinity tolerance have been proposed, including non-stress conditions (Betran et al., 2003), stress conditions, and midway (non-stress and stress) (Ashraf et al., 2015). Selection criteria for salinity stress tolerance include the capacity of germplasm to produce high yields under stress (Rosielle and Hamblin, 1981), the stress susceptibility index being the degree of damage caused (Fischer and Maurer, 1978), and the stress tolerance index, being the percentage of yield under stress and non-stress conditions in the same germplasm (Angessa et al., 2017).The stress tolerance index is not suitable for genotypes that produce low yields under non-stress conditions (Kumar et al., 2014); it can be used to identify genotypes that produce high yields under both stress and non-stress conditions (Askari et al., 2016). Allel et al. (2019) evaluated various indices for salinity tolerance screening and confirmed that the salt-tolerance index is a better selection tool for highly salt-tolerant and productive barley genotypes under salinity, as reported by others (Ali et al., 2007; Shahzad et al., 2012; Senguttuvel et al., 2016). Traits with high rates of variation are among the most indicative and responsive under stress and can be used for the selection using tolerance indices, such as the tolerance index (TOL), salinity susceptibility index (SSI), geometric mean productivity (GMP), mean productivity (MP), and stress tolerance index (STI). Traits with low rates of variation are not suitable for selecting tolerant barley genotypes using tolerance indices under stress (Jamshidi and Javanmard, 2017). Nayyeripasand et al. (2019) reported a positive correlation among stress tolerance indices, including STI, SSI, and TOL, but not in the subgroups. Yu et al. (2018) reported that salt-tolerance levels in rice (*O. sativa*) were not strongly correlated with rice subgroups, which was confirmed in a maize population the following year (Luo et al., 2019). Tolerance indices do not accurately distinguish cultivars under severe stress (Mardeh et al., 2006; Mohammadi, 2019), but can be used as indicators for high-yielding, salt-tolerant lines in stress, and non-stress environments or for traits like germination (Nayyeripasand et al., 2019; Sedri et al., 2019).

### Barley Reference Genome and High-Density Markers Facilitate the Prediction of Candidate Genes Through Genome-Wide Association

To boost barley production in salt-prone areas, unique genes and alleles linked to salt tolerance at germination must be identified in a wider range of barley accessions. GWAS is an alternative and complementary approach that takes advantage of historical recombinations in a high-resolution genome scan to identify regions that are responsive to the traits (Zhao et al., 2011). Several QTLs for salt-tolerant traits at the germination stage have been reported (Mano and Takeda, 1997; Hanen et al., 2014; Angessa et al., 2017). Fan et al. (2015) reported two QTLs for salinity tolerance and N^+^ content on chromosomes 7H and 2H in a DH population of TX9425 × Franklin that were closely linked to markers D7H085710245 and D2H001502476, respectively. A QTL for salinity tolerance mapped on 1H in a YYXT × Franklin DH population was closely linked to marker C1H556900757 (Zhou et al., 2012), as reported in this study. Using 206 barley accessions collected worldwide, 408 Diversity Arrays Technology (DArT) markers, and GWAS, Fan et al. (2016) reported a QTL on 2H that is closely linked to marker D2H001502476. Direct comparisons of our GWAS findings with other studies is tricky, as the marker-trait linkages and chromosomal locations we identified were based on a worldwide barley panel not previously investigated for salinity traits.

Our GWAS for the salinity tolerance index during germination was undertaken on 350 barley accessions using 24,138 DArTseq and SNP markers. Our findings will be a source of new understanding into the genetic basis of salt tolerance at germination and the identification of alleles underlying variation in the trait and candidate genes. Markers with significant effects identified at both locations were selected. We detected 19 QTNs for the tolerance index during germination across the barley genome, showing the complex genetic architecture of salinity tolerance in barley during germination, which is genetically and physiologically controlled by multiple small-effect genes (Flowers, 2004). The significant markers associated with the QTNs will form a basis for marker-assisted selection in barley breeding programs. Conferring with the released genome sequence of barley (Mascher et al., 2017) and gene annotation information, four candidate genes for the tolerance index during germination, belonging to four families, were predicted around the reliable QTNs in the QTN clusters (**Table 6**). In a GWAS study on rice, Naveed et al. (2018) reported 20 QTNs within 22 genes associated with salinity stress at the germination and seedling stages, including kinase family protein, as found in our study. Yu et al. (2018) and Cui et al. (2018) identified 17 and 66 genes, respectively, contributing to salinity tolerance during germination in rice.

Nine markers were identified in Katanning and 12 in Merredin, with four overlapping at both locations (**Supplementary Table 3**). This indicates that salinity tolerance encompasses a complex of mechanisms at both the molecular and plant level that is controlled by many genes affected by the environment and genotype-by-environment (G × E) interactions (Arzani and Ashraf, 2016). The heritability values observed in this study indicated that the variation in tolerance index (germination in salt divided by germination in DI water) was mainly a factor of salinity concentration with a small genetic component. However, salinity tolerance is an important trait in barley that is inherited quantitatively and strongly influenced by environmental conditions (Jabbari et al., 2018), as indicated by the significant interactions among genotypes, salinity tolerance, and location in this study (**Table 1**). Estimated heritability defines how a trait is affected by genotype; however, it is not a total quantifier of how genes and the environment govern a phenotype, but specific to the population and environment under study. It does not account for the effect of missing or the lack of variable factors in the population (Yu et al., 2016).

### Candidate Genes Reveal the Possible Molecular Basis of Salinity Tolerance at Germination

Of the 4 genes associated with salinity stress tolerance traits identified in this study, *P. indica*-insensitive protein 2 is reportedly involved in salinity tolerance through its interaction with phytohormones (auxins, cytokinin, gibberellins, abscisic acid, ethylene, salicylic acid, jasmonates, and brassinosteroids) in *Arabidopsis* (Xu L. et al., 2018). When barley and rice roots were colonized by endophytic basidiomycete fungi (*P. indica*), the host plants enhanced performance under salinity stress (Baltruschat et al., 2008; Vahabi et al., 2016; Jogawat et al., 2016). The protein kinase superfamily is another important gene family that has been characterized in several plants; e.g., for drought tolerance in barley (Cieśla et al., 2016; Yang et al., 2017) and salinity stress tolerance in wheatgrass (Shen et al., 2001). Protein kinase gene family, regulated by transcription factors (TFs) and microRNAs (miRNAs), plays key roles in salt stress tolerance in cotton (Shehzad et al., 2019). Overexpressed transgenic plants of soybean with protein kinase showed significantly increased tolerance to salt stress, suggesting that it plays a pivotal role in salinity tolerance (QIU et al., 2019). *Arabidopsis thaliana*, abscisic acid (ABA)-non-activated protein kinases regulates reactive oxygen species (ROS) homeostasis and triggers genes expression under salinity stress (Szymańska et al., 2019).

In *Arabidopsis*, lipase expression is prompted by NaCl; its overexpression enhances salinity tolerance in transgenic plants, relative to non-transformed control plants, which facilitates seed germination, vegetative growth, flowering, and seed set (Naranjo et al., 2006). Studies have suggested that heat shock protein are likely to be involved in tolerance to other abiotic stresses such as salinity apart from thermal stresses (Song and Ahn, 2011; Mu et al., 2013). Transgenic tobacco plants with heat shock protein of alfalfa exhibited enhanced tolerance to salinity in comparison to wild type plants, in terms of germination rates (Lee et al., 2012). Overexpression of maize heat shock transcription factor enhanced thermo, increased the sensitivity to abscisic acid and salinity stress tolerance in transgenic *Arabidopsis* (Jiang et al., 2018). High expression of heat shock protein genes in barley have been reported in tissue—specific manner salinity stress (Chaudhary et al., 2019). The gene families mentioned above have been associated with stress tolerance, including salinity, in barley, related relatives, and other organisms. This finding will form the basis of more detailed studies to discover and validate the mechanism by which candidate genes play roles in salinity tolerance during germination in barley.

## Data Availability Statement

The datasets analyzed and generated for this study are included in the article/**Supplementary Material**.

## Author Contributions

EM performed phenotyping experiments. EM, GZ, and CH conducted data analysis and interpretation. GZ, X-QZ, and CH generated the genotypic data. TA and CL conducted field experiments. CL, YH, and TA conceived the project. EM drafted paper with inputs from YH, TA, and CH. CL revised the paper and approved the final version for publication.

## Funding

This study was supported by grants from the Australian Grains Research and Development Corporation and Murdoch University through Western Barley Genetic Alliance, Western Australia State Agricultural Biotechnology Centre and The Department of primary and Industry and Regional Development.

## Conflict of Interest

The authors declare that the research was conducted in the absence of any commercial or financial relationships that could be construed as a potential conflict of interest.

## Supplementary Material

Supplementary Figure 1 A map showing geographical representation and origin of barley accessions used in this study.

Supplementary Figure 2 Annual average weather pattern for Merredin and Katanning as reported by the Australian Bureau of Meteorology (http://www.bom.gov.au/index.php).

Supplementary Figure 3 Correlation coefficient for germination in 150 mM NaCl against tolerance index for seeds from Merredin and Katanning.

Supplementary Figure 4 Estimate of the most probable number of clusters (k) and population structure.

Supplementary Figure 5 Average germination percent distribution of 350 barley accession in deionized water.
