# Supplementary figures and images for: Genome-Wide Association Study of Salinity Tolerance During Germination in Barley (Hordeum vulgare L.)
Source: Front Plant Sci. 2020 Feb 21;11:118. doi: 10.3389/fpls.2020.00118 (PMC7047234; doi:10.3389/fpls.2020.00118)

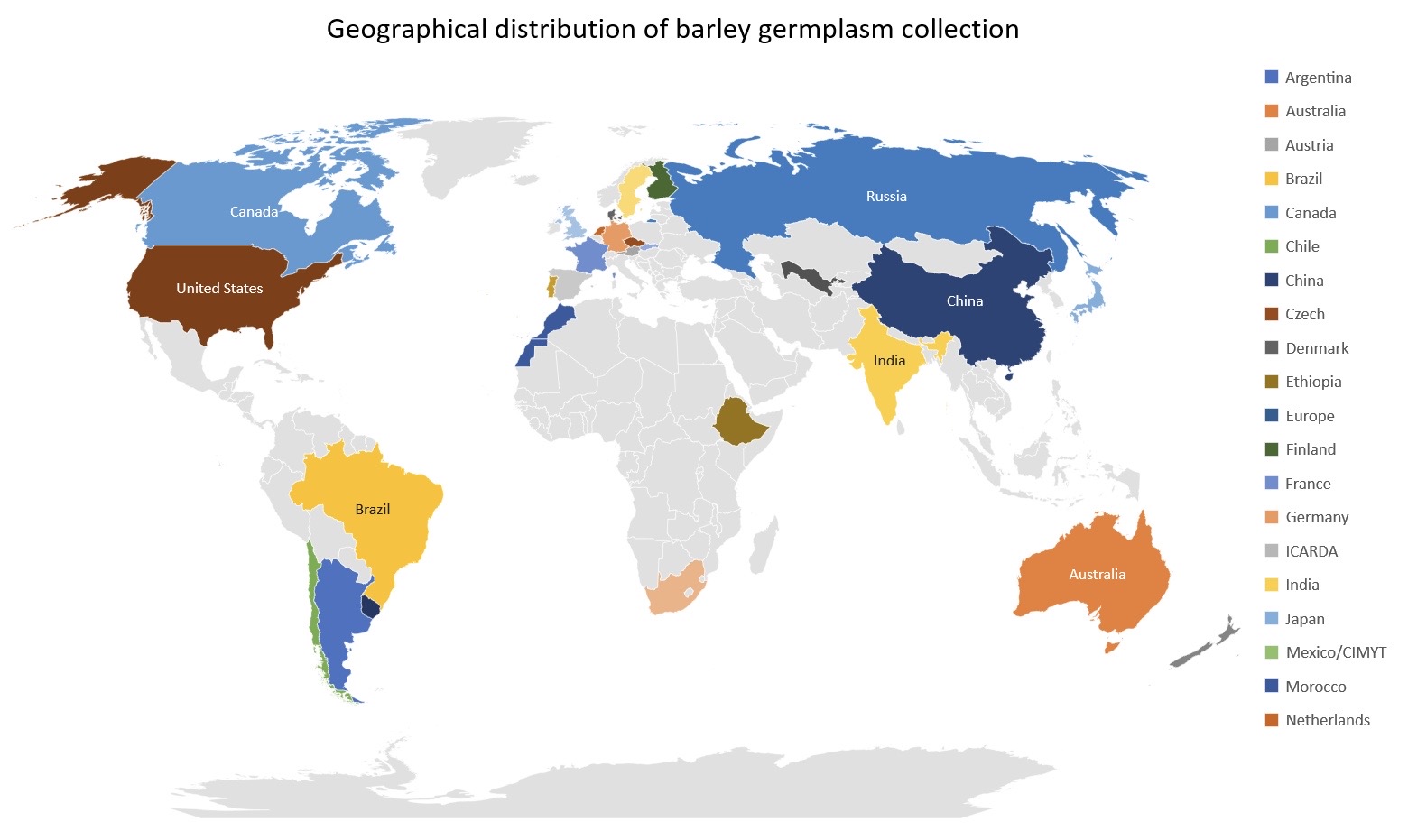

Supplement: Supplementary Figure 1 — A map showing geographical representation and origin of barley accessions used in this study. [file Image_1.jpeg]

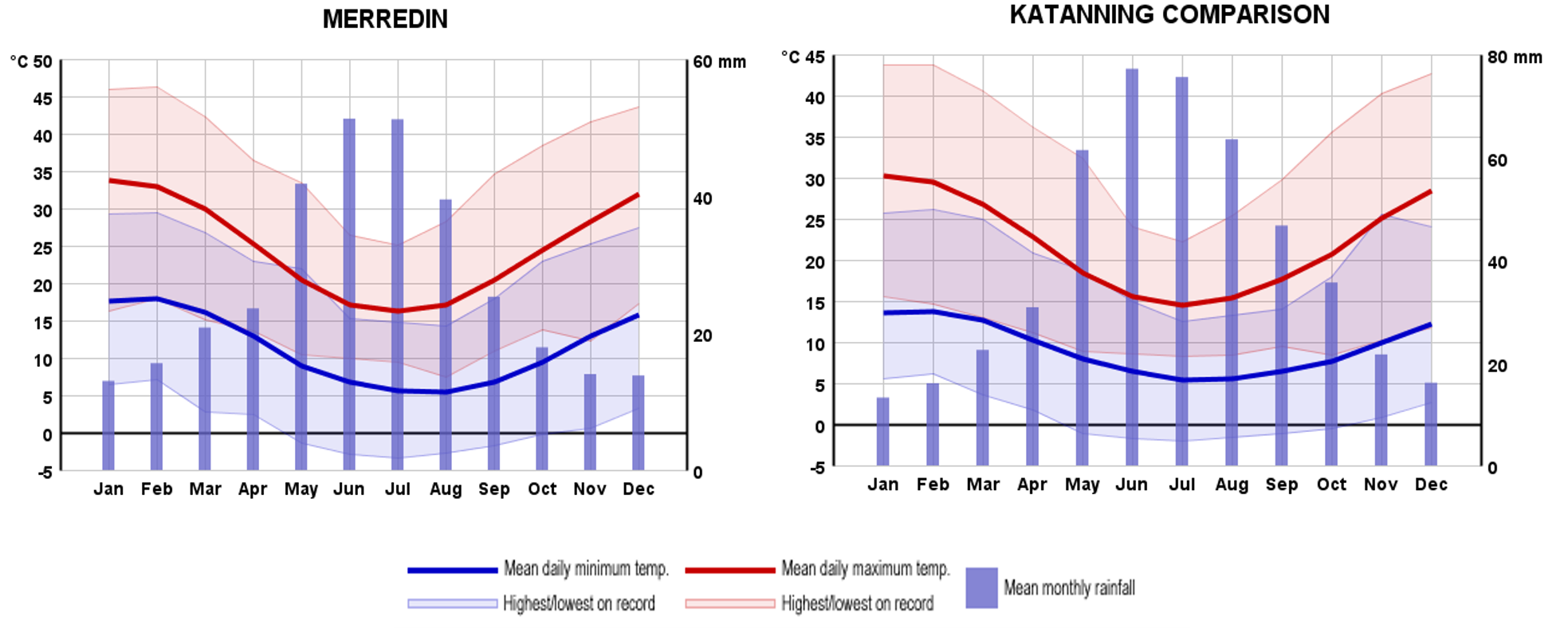

Supplement: Supplementary Figure 2 — Annual average weather pattern for Merredin and Katanning as reported by the Australian Bureau of Meteorology (http://www.bom.gov.au/index.php). [file Image_2.png]

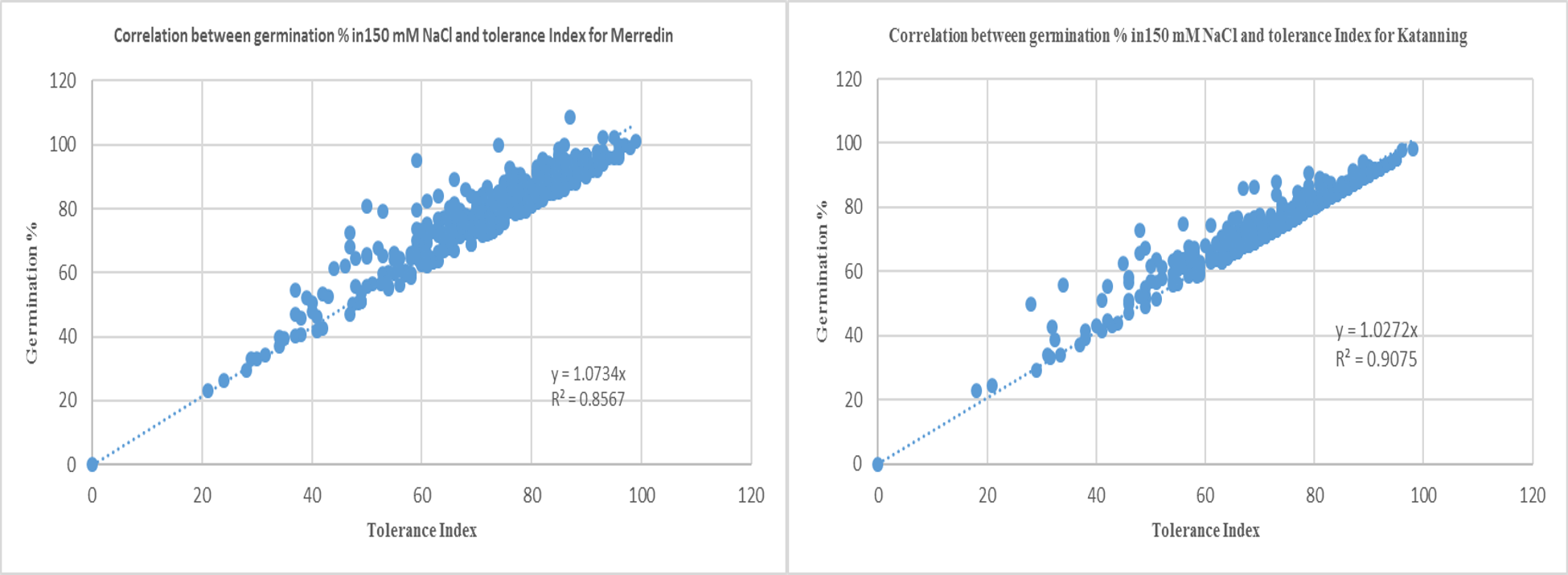

Supplement: Supplementary Figure 3 — Correlation coefficient for germination in 150 mM NaCl against tolerance index for seeds from Merredin and Katanning. [file Image_3.png]

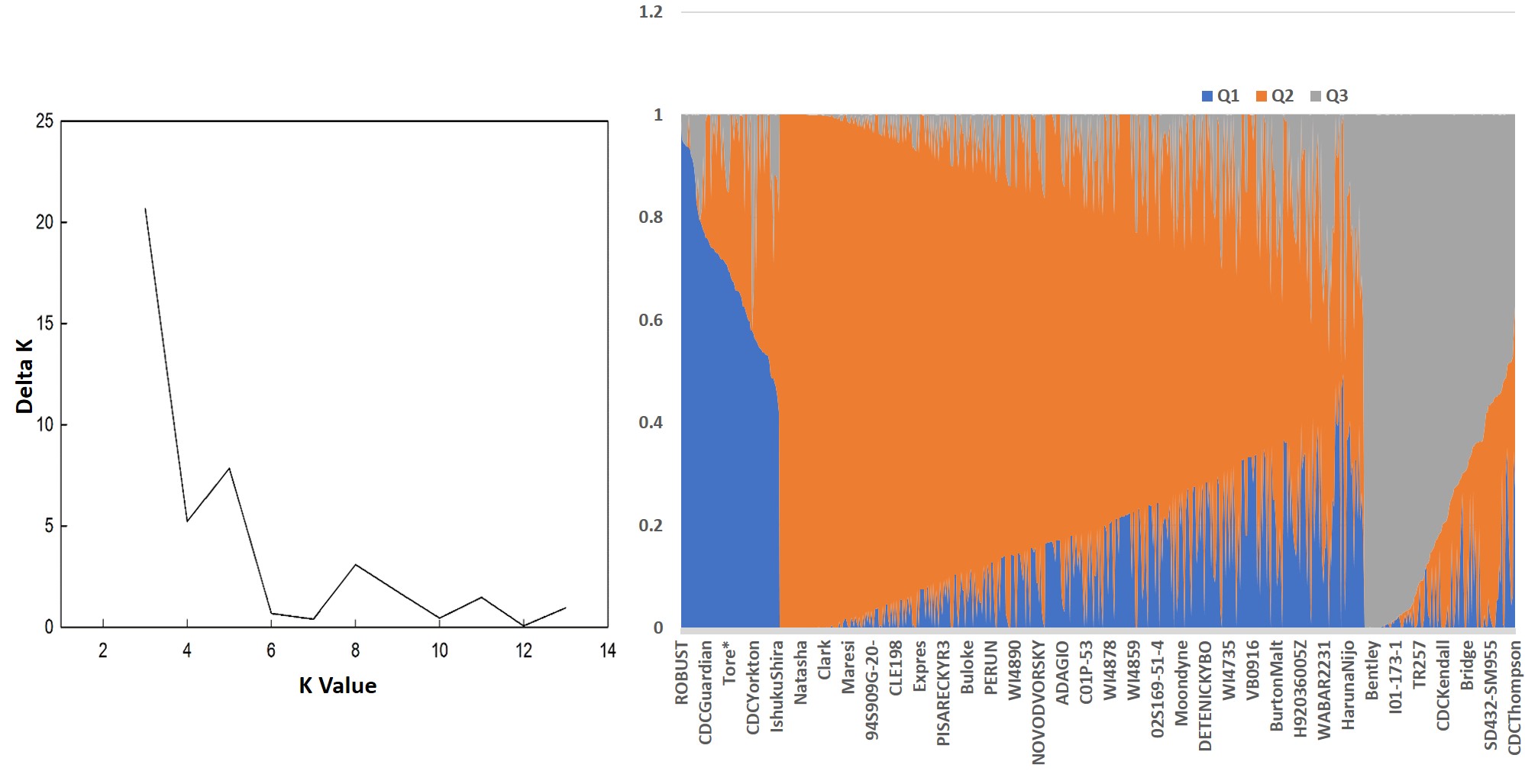

Supplement: Supplementary Figure 4 — Estimate of the most probable number of clusters (k) and population structure. [file Image_4.jpeg]

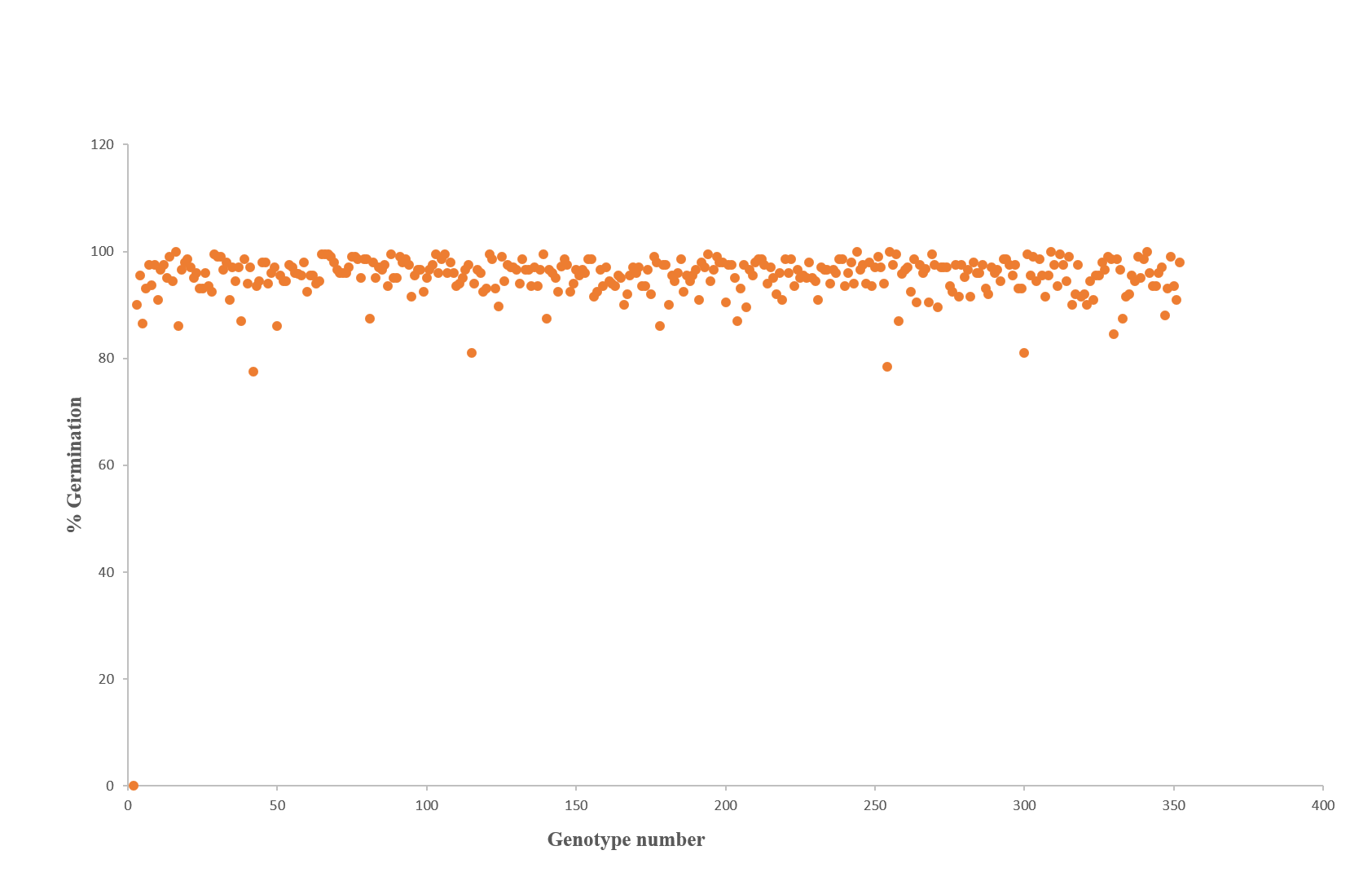

Supplement: Supplementary Figure 5 — Average germination percent distribution of 350 barley accession in deionized water. [file Image_5.png]
