# Supplementary material for: Genome-Wide Association Study of Salinity Tolerance During Germination in Barley (Hordeum vulgare L.)
Source: Front Plant Sci. 2020 Feb 21;11:118. doi: 10.3389/fpls.2020.00118 (PMC7047234; doi:10.3389/fpls.2020.00118)
Supplement: Supplementary file 6 [file Table_1.docx]

**Supplementary Table 1:** Regional representation of barley accessions used in this study

| **Place of origin** | **No. total varieties** | **Percentage** | **No. varieties used** | **Percentage** |
| --- | --- | --- | --- | --- |
| Argentina | 1 | 0.17 | 1 | 0.29 |
| Australia | 211 | 35.52 | 122 | 34.86 |
| Austria | 2 | 0.34 | 1 | 0.29 |
| Brazil | 2 | 0.34 | 1 | 0.29 |
| Canada | 72 | 12.12 | 42 | 12.00 |
| Chile | 1 | 0.17 | 1 | 0.29 |
| China | 7 | 1.18 | 4 | 1.14 |
| Czech | 17 | 2.86 | 10 | 2.86 |
| Denmark | 1 | 0.17 | 1 | 0.29 |
| Ethiopia | 2 | 0.34 | 1 | 0.29 |
| Europe | 12 | 2.02 | 7 | 2.00 |
| Finland | 3 | 0.51 | 2 | 0.57 |
| France | 1 | 0.17 | 1 | 0.29 |
| Germany | 13 | 2.19 | 8 | 2.29 |
| ICARDA | 68 | 11.45 | 39 | 11.14 |
| India | 1 | 0.17 | 1 | 0.29 |
| Japan | 12 | 2.02 | 7 | 2.00 |
| Mexico/CIMMYT | 15 | 2.53 | 9 | 2.57 |
| Morocco | 1 | 0.17 | 1 | 0.29 |
| Netherlands | 2 | 0.34 | 1 | 0.29 |
| New Zealand | 1 | 0.17 | 1 | 0.29 |
| Portugal | 1 | 0.17 | 1 | 0.29 |
| Russia | 2 | 0.34 | 1 | 0.29 |
| Scotland | 2 | 0.34 | 1 | 0.29 |
| Slovakia | 1 | 0.17 | 1 | 0.29 |
| South Africa | 16 | 2.69 | 9 | 2.57 |
| Spain | 1 | 0.17 | 1 | 0.29 |
| Sweden | 3 | 0.51 | 2 | 0.57 |
| UK | 11 | 1.85 | 7 | 2.00 |
| Unknown | 18 | 3.03 | 11 | 3.14 |
| Uruguay | 18 | 3.03 | 11 | 3.14 |
| USA | 74 | 12.46 | 43 | 12.29 |
| Uzbekistan | 2 | 0.34 | 1 | 0.29 |
| **Total** | **594** | **100.00** | 350 | 100. |
