# Supplementary material for: Genome-Wide Association Study of Salinity Tolerance During Germination in Barley (Hordeum vulgare L.)
Source: Front Plant Sci. 2020 Feb 21;11:118. doi: 10.3389/fpls.2020.00118 (PMC7047234; doi:10.3389/fpls.2020.00118)
Supplement: Supplementary file 7 [file Table_2.docx]

**Supplementary Table 2:** List of genotypes used in this study, their origin and growth habits

| **No.** | **Genotype ID** | **Accession name** | **Origin** | **Region** | **Head type** | **Growth habit** |
| --- | --- | --- | --- | --- | --- | --- |
| 1 | P0004 | 02S169-51-45 | AUS | Australia | two-row | spring |
| 2 | P0005 | 04053-034 | AUS | Australia | two-row | spring |
| 3 | P0007 | 04055-119 | AUS | Australia | two-row | spring |
| 4 | P0010 | 04S213D-B-11 | AUS | Australia | two-row | spring |
| 5 | P0014 | 04S213D-B-20 | AUS | Australia | two-row | spring |
| 6 | P0029 | 07T741 | AUS | Australia | two-row | spring |
| 7 | P0035 | 83SL:517 | AUS | Australia | two-row | n.d. |
| 8 | P0036 | 83SM:522 | AUS | Australia | two-row | n.d. |
| 9 | P0038 | 84SL:551 | AUS | Australia | two-row | n.d. |
| 10 | P0039 | 85SW:576 | AUS | Australia | two-row | spring |
| 11 | P0043 | 90S205-129-19 | AUS | Australia | two-row | spring |
| 12 | P0044 | 90S205-45-46 | AUS | Australia | two-row | spring |
| 13 | P0045 | 90S228-151-2 | AUS | Australia | two-row | spring |
| 14 | P0051 | 90SM193-34-32 | AUS | Australia | two-row | spring |
| 15 | P0053 | 91HBSN24 | MEX | North America | two-row | spring |
| 16 | P0054 | 91IBON100 | MEX | North America | two-row | spring |
| 17 | P0057 | 91IBON45 | MEX | North America | six-row | spring |
| 18 | P0058 | 91IBON5 | MEX | North America | two-row | spring |
| 19 | P0062 | 94S909G-20-19 | AUS | Australia | two-row | spring |
| 20 | P0063 | 94S920W-18-6 | AUS | Australia | two-row | spring |
| 21 | P0066 | 95S008-108-27 | AUS | Australia | two-row | spring |
| 22 | P0067 | 95S009-81-33 | AUS | Australia | two-row | spring |
| 23 | P0075 | 96B543 | CAN | North America | two-row | spring |
| 24 | P0084 | AB 47-6 | AUS | Australia | two-row | spring |
| 25 | P0086 | AC METCALFE | CAN | North America | two-row | spring |
| 26 | P0087 | AC Oxbow | CAN | North America | two-row | spring |
| 27 | P0101 | Andre | USA | North America | two-row | spring |
| 28 | P0118 | Atem | GBR | Europe | two-row | spring |
| 29 | P0121 | Atribut | CZE | Europe | two-row | spring |
| 30 | P0127 | B521 | CHN | Asia | two-row | n.d. |
| 31 | P0129 | B559 | CHN | Asia | two-row | n.d. |
| 32 | P0132 | B645 | CHN | Asia | two-row | n.d. |
| 33 | P0133 | B697 | CHN | Asia | two-row | n.d. |
| 34 | P0139 | Barke | DEU | Europe | two-row | spring |
| 35 | P0145 | BEARPAW | USA | North America | two-row | spring |
| 36 | P0146 | Beatrice | FRA | Europe | two-row | spring |
| 37 | P0148 | BEKA | FRA | Europe | two-row | spring |
| 38 | P0149 | Bellini | FRA | Europe | two-row | spring |
| 39 | P0150 | Bentley | CAN | North America | two-row | spring |
| 40 | P0152 | Binalong | AUS | Australia | two-row | spring |
| 41 | P0154 | BM9204-17 | CAN | North America | two-row | spring |
| 42 | P0155 | BM9311-35 | CAN | North America | two-row | spring |
| 43 | P0156 | BM9507-109 | CAN | North America | two-row | spring |
| 44 | P0158 | BM9645-96 | CAN | North America | two-row | spring |
| 45 | P0159 | BM9647D-43 | CAN | North America | two-row | spring |
| 46 | P0160 | BM9647D-66 | CAN | North America | two-row | spring |
| 47 | P0161 | BM9752D-125 | CAN | North America | two-row | spring |
| 48 | P0162 | BM9857-263-1 | CAN | North America | two-row | spring |
| 49 | P0167 | BoaFe | PRT | Europe | two-row | spring |
| 50 | P0169 | BOLRON | USA | North America | six-row | facultative |
| 51 | P0171 | BOWMAN | USA | North America | two-row | spring |
| 52 | P0175 | Braemar | GBR | Europe | two-row | spring |
| 53 | P0177 | Bridge | CAN | North America | two-row | spring |
| 54 | P0178 | Brindabella | AUS | Australia | two-row | spring |
| 55 | P0180 | BT558 | CAN | North America | six-row | winter |
| 56 | P0181 | BT634-AC Lacombe | CAN | North America | six-row | spring |
| 57 | P0183 | Buloke | AUS | Australia | two-row | spring |
| 58 | P0184 | Burton Malt | GBR | Europe | two-row | spring |
| 59 | P0187 | BVDV-026 | MEX | North America | two-row | n.d. |
| 60 | P0190 | C01P-37 | URY | South America | two-row | n.d. |
| 61 | P0191 | C01P-53 | URY | South America | two-row | n.d. |
| 62 | P0192 | C01P-66 | URY | South America | two-row | n.d. |
| 63 | P0194 | C04A-34 | URY | South America | two-row | n.d. |
| 64 | P0196 | C2-05-10/263 | URY | South America | two-row | n.d. |
| 65 | P0197 | C2-05-10/437 | URY | South America | two-row | n.d. |
| 66 | P0199 | C2-05-337-2 | AUS | Australia | two-row | spring |
| 67 | P0201 | C2-05-63/710 | URY | South America | two-row | n.d. |
| 68 | P0203 | C2-05-89/827 | URY | South America | two-row | n.d. |
| 69 | P0205 | C2-05-89/878 | URY | South America | two-row | n.d. |
| 70 | P0210 | C98Prel-29 | URY | South America | two-row | n.d. |
| 71 | P0212 | C98Prel-60 | URY | South America | two-row | n.d. |
| 72 | P0226 | CBSS98M00022T-0TOPY-0M-1Y-2M-0Y | MEX | North America | six-row | spring |
| 73 | P0230 | CDC Dolly | CAN | North America | two-row | spring |
| 74 | P0233 | CDC Guardian | CAN | North America | two-row | spring |
| 75 | P0237 | CDC Meredith | CAN | North America | two-row | spring |
| 76 | P0245 | CDC Thompson | CAN | North America | two-row | spring |
| 77 | P0242 | CDC TISDALE | CAN | North America | six-row | spring |
| 78 | P0243 | CDC UNITY | CAN | North America | two-row | spring |
| 79 | P0244 | CDC Yorkton | CAN | North America | six-row | spring |
| 80 | P0250 | Chapais | CAN | North America | six-row | spring |
| 81 | P0251 | Charger | AUS | Australia | two-row | spring |
| 82 | P0253 | Charlottetown | CAN | North America | two-row | spring |
| 83 | P0255 | CHERI | DEU | Europe | two-row | spring |
| 84 | P0261 | CI5791 | ETH | Africa | two-row | spring |
| 85 | P0262 | CI9819 | ETH | Africa | two-row | spring |
| 86 | P0266 | Clark | USA | North America | two-row | spring |
| 87 | P0275 | CLE235 | URY | South America | two-row | spring |
| 88 | P0277 | CLE268 | URY | South America | two-row | spring |
| 89 | P0278 | CLE270 | URY | South America | two-row | spring |
| 90 | P0282 | CM67 | USA | North America | six-row | spring |
| 91 | P0284 | Commander | AUS | Australia | two-row | spring |
| 92 | P0285 | Compass | AUS | Australia | two-row | spring |
| 93 | P0291 | CORGI | GBR | Europe | two-row | spring |
| 94 | P0292 | Cowabbie | AUS | Australia | two-row | spring |
| 95 | P0303 | Defra | DEU | Europe | two-row | spring |
| 96 | P0307 | Derkado | GBR | Europe | two-row | spring |
| 97 | P0309 | DH29287 | AUS | Australia | two-row | spring |
| 98 | P0313 | DH29400 | AUS | Australia | two-row | spring |
| 99 | P0316 | DIABAS | CZE | Europe | two-row | spring |
| 100 | P0324 | DVORAN | SVK | Europe | two-row | spring |
| 101 | P0325 | E Dong 85-1 | CHN | Asia | two-row | spring |
| 102 | P0327 | EB1111 | AUS | Australia | two-row | spring |
| 103 | P0330 | EMIRCOMPLEX | SWE | Europe | two-row | spring |
| 104 | P0332 | ESPERANCE ORGE 289 | n.d. | n.d. | two-row | spring |
| 105 | P0334 | EUROPA | NLD | Europe | two-row | spring |
| 106 | P0337 | Fairview | NZL | Australia | two-row | spring |
| 107 | P0342 | FILIPPA | SWE | Europe | two-row | spring |
| 108 | P0343 | Finniss | AUS | Australia | two-row | spring |
| 109 | P0346 | Flagon | GBR | Europe | two-row | winter |
| 110 | P0347 | Flagship | AUS | Australia | two-row | spring |
| 111 | P0348 | Fleet | AUS | Australia | two-row | spring |
| 112 | P0349 | Flinders | AUS | Australia | two-row | spring |
| 113 | P0350 | FORMULA | GBR | Europe | two-row | spring |
| 114 | P0352 | Foster | USA | North America | six-row | spring |
| 115 | P0353 | FR/DAYTON | USA | North America | six-row | winter |
| 116 | P0355 | France 30161 | FRA | Europe | two-row | winter |
| 117 | P0361 | GALAN | CZE | Europe | two-row | spring |
| 118 | P0371 | GrangeR | AUS | Australia | two-row | spring |
| 119 | P0372 | Granifen | CZE | Europe | two-row | spring |
| 120 | P0373 | Grimmett | AUS | Australia | two-row | spring |
| 121 | P0374 | Grout | AUS | Australia | two-row | spring |
| 122 | P0376 | GSHO 2483 (in XV2334-6R from Indian Dwarf, slender dwarf 5 mutant) | USA | North America | six-row | spring |
| 123 | P0379 | H92014002X | CAN | North America | two-row | winter |
| 124 | P0380 | H92036005Z | CAN | North America | two-row | winter |
| 125 | P0383 | H96009006 | CAN | North America | six-row | winter |
| 126 | P0386 | Hamelin | AUS | Australia | two-row | spring |
| 127 | P0389 | Hannan | AUS | Australia | two-row | spring |
| 128 | P0390 | HANNCHEN | SWE | Europe | two-row | spring |
| 129 | P0391 | Har.Nan-35-24 | AUS | Australia | two-row | spring |
| 130 | P0392 | Har.Nan-35-28 | AUS | Australia | two-row | spring |
| 131 | P0394 | HARRINGTON | CAN | North America | two-row | spring |
| 132 | P0398 | Haruna Nijo | JPN | Asia | two-row | spring |
| 133 | P0399 | Hassan | NLD | Europe | two-row | spring |
| 134 | P0400 | Havanna | CZE | Europe | two-row | spring |
| 135 | P0401 | HB08306 | CAN | North America | two-row | spring |
| 136 | P0402 | HB09309 | CAN | North America | two-row | spring |
| 137 | P0404 | HB344-SB93666 | CAN | North America | two-row | spring |
| 138 | P0405 | HB352 | CAN | North America | two-row | spring |
| 139 | P0407 | HB380 | CAN | North America | two-row | spring |
| 140 | P0408 | HB382 | CAN | North America | two-row | spring |
| 141 | P0409 | HB385 | CAN | North America | two-row | spring |
| 142 | P0410 | HB395 | CAN | North America | two-row | spring |
| 143 | P0412 | HB805-BZ594-26 | CAN | North America | two-row | spring |
| 144 | P0413 | Heart | GBR | Europe | two-row | spring |
| 145 | P0417 | Heriot | GBR | Europe | two-row | spring |
| 146 | P0418 | Heris | CZE | Europe | two-row | spring |
| 147 | P0419 | Hindmarsh | AUS | Australia | two-row | spring |
| 148 | P0424 | I01-106-2-2 | USA | North America | two-row | n.d. |
| 149 | P0425 | I01-173-1 | USA | North America | two-row | n.d. |
| 150 | P0428 | I01-179-4 | USA | North America | two-row | n.d. |
| 151 | P0429 | I01-302-1 | USA | North America | two-row | n.d. |
| 152 | P0435 | I90-137-1 | USA | North America | two-row | spring |
| 153 | P0436 | I91-454 | USA | North America | two-row | spring |
| 154 | P0437 | I91-495 | USA | North America | two-row | spring |
| 155 | P0440 | I91-696 | USA | North America | two-row | spring |
| 156 | P0445 | I93-608 | USA | North America | two-row | spring |
| 157 | P0451 | I97-415 | USA | North America | two-row | spring |
| 158 | P0453 | ICB 104039 | AFG | Middle East | six-row | facultative |
| 159 | P0454 | ICB78-0058-7AP-2AP-1AP-4AP-0AP | SYR | Middle East | two-row | spring |
| 160 | P0456 | IG 16957 | UZB | Asia | six-row | spring |
| 161 | P0460 | IGB1120 | AUS | Australia | two-row | spring |
| 162 | P0462 | IGB1133 | AUS | Australia | two-row | spring |
| 163 | P0463 | IGB1138 | AUS | Australia | two-row | spring |
| 164 | P0469 | IGB1234 | AUS | Australia | two-row | spring |
| 165 | P0471 | IGB1243 | AUS | Australia | two-row | spring |
| 166 | P0472 | IGB1244 | AUS | Australia | two-row | spring |
| 167 | P0475 | IGV3-313 (ATLAS46) | USA | North America | six-row | spring |
| 168 | P0476 | Ilka | DEU | Europe | two-row | spring |
| 169 | P0477 | Inari | FIN | Europe | two-row | spring |
| 170 | P0479 | Ishuku Shirazu | JPN | Asia | two-row | winter |
| 171 | P0480 | Jantar | CZE | Europe | two-row | spring |
| 172 | P0482 | Jubilant | SVK | Europe | two-row | spring |
| 173 | P0483 | Jyoti-PI 428399 | IND | Asia | six-row | spring |
| 174 | P0484 | Kalkreuther Fruhe | DEU | Europe | six-row | winter |
| 175 | P0485 | Kaputar | AUS | Australia | two-row | spring |
| 176 | P0490 | Keel | AUS | Australia | two-row | spring |
| 177 | P0495 | Kinukei 18 | JPN | Asia | two-row | spring |
| 178 | P0496 | Kinukei 19 | JPN | Asia | two-row | spring |
| 179 | P0499 | KLAXON | GBR | Europe | two-row | spring |
| 180 | P0505 | KRYSTAL (Mla13) | YUG | Europe | two-row | winter |
| 181 | P0507 | Kustaa | SWE | Europe | two-row | spring |
| 182 | P0513 | Landlord | GBR | Europe | two-row | spring |
| 183 | P0517 | Larker | USA | North America | six-row | spring |
| 184 | P0518 | Legacy | USA | North America | six-row | spring |
| 185 | P0521 | Lindwall | AUS | Australia | two-row | spring |
| 186 | P0525 | LOCEB-30 | MEX | North America | two-row | spring |
| 187 | P0526 | Lockyer | AUS | Australia | two-row | spring |
| 188 | P0531 | Luxor | CZE | Europe | six-row | winter |
| 189 | P0537 | Mackay | AUS | Australia | two-row | spring |
| 190 | P0538 | Macquarie | AUS | Australia | two-row | spring |
| 191 | P0541 | Maltine | NLD | Europe | two-row | spring |
| 192 | P0544 | Maresi | DEU | Europe | two-row | spring |
| 193 | P0546 | Maritime | AUS | Australia | two-row | spring |
| 194 | P0550 | MC9924-012 | CAN | North America | two-row | winter |
| 195 | P0552 | MC9924-031 | CAN | North America | two-row | winter |
| 196 | P0553 | MC9939-007 | CAN | North America | two-row | winter |
| 197 | P0555 | MC9939-016 | CAN | North America | two-row | winter |
| 198 | P0557 | MC9939-039 | CAN | North America | two-row | winter |
| 199 | P0558 | MC9939-048 | CAN | North America | two-row | winter |
| 200 | P0563 | MN599 | BRA | South America | two-row | spring |
| 201 | P0564 | MN607 | BRA | South America | two-row | spring |
| 202 | P0570 | Moondyne | AUS | Australia | two-row | spring |
| 203 | P0572 | Morex | USA | North America | six-row | spring |
| 204 | P0574 | Moroc9-75/ArabiAswad/4/Hml-02/ArabiAbiad/3/Api/CM67//Nacta | SYR | Middle East | two-row | n.d. |
| 205 | P0575 | MoroccanLandrace | MAR | Africa | six-row | spring |
| 206 | P0573 | Morovian | USA | North America | six-row | spring |
| 207 | P0576 | Mosane | BEL | Europe | two-row | spring |
| 208 | P0581 | Natasha | FRA | Europe | two-row | spring |
| 209 | P0583 | NB1054/ALELI | MEX | North America | six-row | spring |
| 210 | P0584 | NBX05019-08-099 | AUS | Australia | two-row | spring |
| 211 | P0587 | ND22170 | USA | North America | two-row | n.d. |
| 212 | P0588 | ND23265 | USA | North America | two-row | n.d. |
| 213 | P0589 | ND23275 | USA | North America | two-row | n.d. |
| 214 | P0592 | Newdale | CAN | North America | two-row | spring |
| 215 | P0594 | Nirasaki Nijo 9 | JPN | Asia | two-row | spring |
| 216 | P0602 | NRB08308 | AUS | Australia | two-row | spring |
| 217 | P0606 | O'Connor | AUS | Australia | two-row | spring |
| 218 | P0613 | Optic | GBR | Europe | two-row | spring |
| 219 | P0615 | ORBIT | SVK | Europe | two-row | spring |
| 220 | P0616 | OTIS | USA | North America | two-row | spring |
| 221 | P0619 | Oxford | AUS | Australia | two-row | spring |
| 222 | P0621 | Patty | FRA | Europe | two-row | spring |
| 223 | P0622 | Pearl | GBR | Europe | two-row | winter |
| 224 | P0624 | PERUN | CZE | Europe | two-row | spring |
| 225 | P0625 | PEWTER | DNK | Europe | two-row | spring |
| 226 | P0626 | PICCOLO | NLD | Europe | two-row | spring |
| 227 | P0635 | Prosa | AUT | Europe | two-row | spring |
| 228 | P0638 | Quasar | AUS | Australia | two-row | spring |
| 229 | P0641 | Rawson | USA | North America | two-row | spring |
| 230 | P0645 | Regina | DEU | Europe | two-row | winter |
| 231 | P0646 | RETROARUPOBV-9225 | MEX | North America | two-row | spring |
| 232 | P0648 | Riviera | GBR | Europe | two-row | spring |
| 233 | P0650 | Roe | AUS | Australia | two-row | spring |
| 234 | P0655 | RUSSIA24 | RUS | Europe | two-row | spring |
| 235 | P0657 | Ruti | CZE | Europe | two-row | spring |
| 236 | P0661 | SANALTA | CAN | North America | two-row | spring |
| 237 | P0662 | Satsuki Nijo | JPN | Asia | two-row | spring |
| 238 | P0663 | SB01513 | GBR | Europe | two-row | spring |
| 239 | P0664 | SB03180 | GBR | Europe | two-row | spring |
| 240 | P0667 | SB99252 | GBR | Europe | two-row | spring |
| 241 | P0670 | Schooner | AUS | Australia | two-row | spring |
| 242 | P0674 | SE612.01 | DEU | Europe | two-row | n.d. |
| 243 | P0676 | SEEBE | MEX | North America | two-row | spring |
| 244 | P0679 | SH040468 | CAN | North America | two-row | winter |
| 245 | P0682 | Shepherd | AUS | Australia | two-row | spring |
| 246 | P0684 | Shinonome | JPN | Asia | six-row | spring |
| 247 | P0685 | SHN094 | USA | North America | two-row | n.d. |
| 248 | P0687 | SHYRI | MEX | North America | two-row | spring |
| 249 | P0689 | Sissy | DEU | Europe | two-row | spring |
| 250 | P0690 | Skiff | AUS | Australia | two-row | spring |
| 251 | P0692 | Sloop | AUS | Australia | two-row | spring |
| 252 | P0693 | Sloop SA | AUS | Australia | two-row | spring |
| 253 | P0694 | Sloop VIC | AUS | Australia | two-row | spring |
| 254 | P0697 | SM02544 | CAN | North America | two-row | winter |
| 255 | P0698 | SM060103 | CAN | North America | two-row | winter |
| 256 | P0703 | SMBA11-1771 | AUS | Australia | two-row | spring |
| 257 | P0709 | SpanishLandrace-355 | ESP | Europe | n.d. | n.d. |
| 258 | P0714 | Steffi | DEU | Europe | two-row | spring |
| 259 | P0715 | Stella | SWE | Europe | two-row | spring |
| 260 | P0716 | Stellar-ND | USA | North America | six-row | spring |
| 261 | P0718 | Stirling | AUS | Australia | two-row | spring |
| 262 | P0720 | Sublette | USA | North America | two-row | spring |
| 263 | P0723 | SVB21 | ZAF | Africa | two-row | n.d. |
| 264 | P0724 | SVB24 (lfrR) | ZAF | Africa | two-row | n.d. |
| 265 | P0726 | SVC5 | ZAF | Africa | two-row | n.d. |
| 266 | P0728 | Syn6058-06 | AUS | Australia | two-row | spring |
| 267 | P0730 | Tallon | AUS | Australia | two-row | spring |
| 268 | P0731 | TANKARD | CAN | North America | two-row | spring |
| 269 | P0732 | Tantangara | AUS | Australia | two-row | spring |
| 270 | P0736 | Tilga | AUS | Australia | two-row | spring |
| 271 | P0737 | Tipper//WI2291/WI2269 | SYR | Middle East | two-row | spring |
| 272 | P0739 | Tocada | DEU | Europe | two-row | spring |
| 273 | P0740 | Toddy | GBR | Europe | two-row | spring |
| 274 | P0743 | Tore* | NOR | Europe | two-row | spring |
| 275 | P0744 | Torrens | AUS | Australia | two-row | spring |
| 276 | P0749 | TR06390 | CAN | North America | two-row | spring |
| 277 | P0751 | TR07393 | CAN | North America | two-row | spring |
| 278 | P0756 | TR117a | CAN | North America | two-row | spring |
| 279 | P0757 | TR145 | CAN | North America | two-row | spring |
| 280 | P0762 | TR245 | CAN | North America | two-row | spring |
| 281 | P0763 | TR257 | CAN | North America | two-row | spring |
| 282 | P0768 | TR638 | CAN | North America | two-row | spring |
| 283 | P0769 | TR645 | CAN | North America | two-row | spring |
| 284 | P0777 | Tulla | AUS | Australia | two-row | spring |
| 285 | P0779 | Unicorn | JPN | Asia | two-row | spring |
| 286 | P0781 | Urambie | AUS | Australia | two-row | winter |
| 287 | P0782 | Ursa | CZE | Europe | two-row | spring |
| 288 | P0786 | UWA94TK18-18 | AUS | Australia | two-row | spring |
| 289 | P0788 | UWA96T45-07-26 | AUS | Australia | two-row | spring |
| 290 | P0794 | Valeta | NLD | Europe | two-row | spring |
| 291 | P0796 | VB0330 | AUS | Australia | two-row | spring |
| 292 | P0800 | VB0904 | AUS | Australia | two-row | spring |
| 293 | P0801 | VB0916 | AUS | Australia | two-row | spring |
| 294 | P0806 | VIC--8717 | AUS | Australia | two-row | spring |
| 295 | P0809 | Vlamingh | AUS | Australia | two-row | spring |
| 296 | P0810 | VODKA | FRA | Europe | two-row | spring |
| 297 | P0813 | VT Admiral | AUS | Australia | two-row | spring |
| 298 | P0814 | W2 2010-5-17 | AUS | Australia | six-row | spring |
| 299 | P0817 | WABAR2228 | AUS | Australia | two-row | spring |
| 300 | P0818 | WABAR2231 | AUS | Australia | two-row | spring |
| 301 | P0819 | WABAR2234 | AUS | Australia | two-row | spring |
| 302 | P0825 | WABAR2259 | AUS | Australia | two-row | spring |
| 303 | P0830 | WABAR2347 | AUS | Australia | two-row | spring |
| 304 | P0832 | WABAR2377 | AUS | Australia | two-row | spring |
| 305 | P0833 | WABAR2378 | AUS | Australia | two-row | spring |
| 306 | P0834 | WABAR2411 | AUS | Australia | two-row | spring |
| 307 | P0835 | WABAR2421 | AUS | Australia | two-row | spring |
| 308 | P0837 | WABAR2425 | AUS | Australia | two-row | spring |
| 309 | P0841 | WABAR2547 | AUS | Australia | two-row | spring |
| 310 | P0843 | WABAR2592 | AUS | Australia | two-row | spring |
| 311 | P0844 | WABAR2609 | AUS | Australia | two-row | spring |
| 312 | P0854 | WB146 | AUS | Australia | two-row | spring |
| 313 | P0858 | WI2553 | AUS | Australia | two-row | spring |
| 314 | P0860 | WI2816 | AUS | Australia | two-row | spring |
| 315 | P0862 | WI2868 | AUS | Australia | two-row | spring |
| 316 | P0883 | WI4574 | AUS | Australia | two-row | spring |
| 317 | P0885 | WI4584 | AUS | Australia | two-row | spring |
| 318 | P0887 | WI4597 | AUS | Australia | two-row | spring |
| 319 | P0889 | WI4619 | AUS | Australia | two-row | spring |
| 320 | P0891 | WI4638 | AUS | Australia | two-row | spring |
| 321 | P0897 | WI4666 | AUS | Australia | two-row | spring |
| 322 | P0899 | WI4683 | AUS | Australia | two-row | spring |
| 323 | P0901 | WI4704 | AUS | Australia | two-row | spring |
| 324 | P0905 | WI4715 | AUS | Australia | two-row | spring |
| 325 | P0912 | WI4741 | AUS | Australia | two-row | spring |
| 326 | P0920 | WI4768 | AUS | Australia | two-row | spring |
| 327 | P0924 | WI4801 | AUS | Australia | two-row | spring |
| 328 | P0925 | WI4843 | AUS | Australia | two-row | spring |
| 329 | P0926 | WI4847 | AUS | Australia | two-row | spring |
| 330 | P0927 | WI4849 | AUS | Australia | two-row | spring |
| 331 | P0928 | WI4854 | AUS | Australia | two-row | spring |
| 332 | P0931 | WI4870 | AUS | Australia | two-row | spring |
| 333 | P0932 | WI4874 | AUS | Australia | two-row | spring |
| 334 | P0933 | WI4876 | AUS | Australia | two-row | spring |
| 335 | P0935 | WI4879 | AUS | Australia | two-row | spring |
| 336 | P0936 | WI4882 | AUS | Australia | two-row | spring |
| 337 | P0937 | WI4885 | AUS | Australia | two-row | spring |
| 338 | P0938 | WI4886 | AUS | Australia | two-row | spring |
| 339 | P0941 | WI4890 | AUS | Australia | two-row | spring |
| 340 | P0943 | WI4893 | AUS | Australia | two-row | spring |
| 341 | P0944 | Wicket | GBR | Europe | two-row | spring |
| 342 | P0951 | WVA22 | ZAF | Africa | two-row | n.d. |
| 343 | P0952 | WVB35 | ZAF | Africa | two-row | n.d. |
| 344 | P0953 | WVC3 | ZAF | Africa | two-row | n.d. |
| 345 | P0959 | XVE7 | ZAF | Africa | two-row | n.d. |
| 346 | P0960 | XVH11 | ZAF | Africa | two-row | n.d. |
| 347 | P0962 | Yambla | AUS | Australia | two-row | spring |
| 348 | P0967 | Z019Q008R | USA | North America | two-row | n.d. |
| 349 | P0972 | ZBC9322 | MEX | North America | two-row | spring |
| 350 | P0974 | ZBC934 | MEX | North America | two-row | spring |
