# Supplementary material for: Genome-Wide Association Study of Salinity Tolerance During Germination in Barley (Hordeum vulgare L.)
Source: Front Plant Sci. 2020 Feb 21;11:118. doi: 10.3389/fpls.2020.00118 (PMC7047234; doi:10.3389/fpls.2020.00118)
Supplement: Supplementary file 8 [file Table_3.docx]

| **Supplementary Table 3:** Genome wide association analysis for salinity tolerance index at germination stage | | | | | | |
| --- | --- | --- | --- | --- | --- | --- |
| **Location** | **Marker** | **Chr** | **Position (bp)** | **MarkerR2** | **–log10 (P)** | **q-FDR** |
| Merredin | L1H018492689 | H1 | 18492689 | 0.087 | 5.514 | 0.028 |
|  | L1H018492798 | H1 | 18492798 | 0.090 | 5.437 | 0.028 |
|  | L1H018495748 | H1 | 18495748 | 0.087 | 5.300 | 0.039 |
|  | D1H528333687 | H1 | 528333687 | 0.083 | 4.982 | 0.045 |
|  | C1H556900705 | H1 | 556900705 | 0.082 | 5.402 | 0.028 |
|  | C1H556900787 | H1 | 556900787 | 0.077 | 5.277 | 0.038 |
|  | D2H001502476 | H2 | 1502476 | 0.087 | 4.864 | 0.048 |
|  | D3H598501321 | H3 | 598501321 | 0.079 | 4.869 | 0.048 |
|  | L4H635824216 | H4 | 635824216 | 0.075 | 4.676 | 0.050 |
|  | L5H044127079 | H5 | 44127079 | 0.082 | 4.941 | 0.047 |
|  | L6H286731484 | H6 | 286731484 | 0.074 | 4.261 | 0.071 |
|  | D6H471369639 | H6 | 471369639 | 0.067 | 4.324 | 0.064 |
|  | L6H495910722 | H6 | 495910722 | 0.078 | 4.722 | 0.049 |
|  | D7H016569501 | H7 | 16569501 | 0.063 | 4.049 | 0.088 |
|  | D7H085710245 | H7 | 85710245 | 0.079 | 4.420 | 0.054 |
|  | L7H212035410 | H7 | 212035410 | 0.079 | 4.730 | 0.049 |
|  | L7H614807240 | H7 | 614807240 | 0.072 | 4.425 | 0.053 |
|  | D7H638672485 | H7 | 638672485 | 0.066 | 4.241 | 0.076 |
| Katanning | L1H018492689 | H1 | 18492689 | 0.102 | 5.934 | 0.018 |
|  | L1H018492798 | H1 | 18492798 | 0.100 | 5.889 | 0.019 |
|  | D1H528333687 | H1 | 528333687 | 0.088 | 4.915 | 0.036 |
|  | L2H525371651 | H2 | 525371651 | 0.096 | 4.893 | 0.037 |
|  | L5H070630348 | H5 | 70630348 | 0.082 | 4.841 | 0.037 |
|  | L6H002587116 | H6 | 2587116 | 0.085 | 4.722 | 0.039 |
|  | L6H004005746 | H6 | 4005746 | 0.087 | 4.878 | 0.037 |
|  | D6H074421386 | H6 | 74421386 | 0.073 | 4.482 | 0.051 |
|  | L7H004015622 | H7 | 4015622 | 0.099 | 5.443 | 0.020 |
|  | D7H085710245 | H7 | 85710245 | 0.071 | 4.403 | 0.059 |
|  | L7H212035410 | H7 | 212035410 | 0.114 | 5.816 | 0.019 |
|  | C7H653619080 | H7 | 653619080 | 0.067 | 4.139 | 0.074 |
|  | D7H655103370 | H7 | 655103370 | 0.085 | 5.312 | 0.029 |
| Average | L1H018492689 | H1 | 18492689 | 0.110 | 6.352 | 0.006 |
|  | L1H018492798 | H1 | 18492798 | 0.110 | 6.172 | 0.006 |
|  | L1H021222161 | H1 | 21222161 | 0.095 | 5.263 | 0.013 |
|  | D1H528333687 | H1 | 528333687 | 0.097 | 5.440 | 0.013 |
|  | D2H001502476 | H2 | 1502476 | 0.090 | 4.961 | 0.030 |
|  | D3H598501321 | H3 | 598501321 | 0.100 | 6.357 | 0.006 |
|  | L3H687771598 | H3 | 687771598 | 0.089 | 5.086 | 0.029 |
|  | L4H007417825 | H4 | 7417825 | 0.082 | 5.215 | 0.016 |
|  | L5H017667933 | H5 | 17667933 | 0.090 | 4.893 | 0.030 |
|  | L5H044127079 | H5 | 44127079 | 0.086 | 5.289 | 0.028 |
|  | L5H232131131 | H5 | 232131131 | 0.087 | 4.626 | 0.035 |
|  | L6H015979347 | H6 | 15979347 | 0.075 | 4.553 | 0.043 |
|  | L6H042597682 | H6 | 42597682 | 0.073 | 4.310 | 0.051 |
|  | L6H042597693 | H6 | 42597693 | 0.073 | 4.396 | 0.050 |
|  | L6H286731484 | H6 | 286731484 | 0.080 | 4.947 | 0.023 |
|  | L6H495910722 | H6 | 495910722 | 0.079 | 4.589 | 0.043 |
|  | L6H502983510 | H6 | 502983510 | 0.075 | 4.480 | 0.050 |
|  | L7H004015622 | H7 | 4015622 | 0.083 | 4.984 | 0.030 |
|  | D7H085710245 | H7 | 85710245 | 0.082 | 4.845 | 0.030 |
|  | L7H212035410 | H7 | 212035410 | 0.100 | 5.802 | 0.009 |
|  | L7H614807240 | H7 | 614807240 | 0.075 | 4.496 | 0.050 |
