# Supplementary material for: Genome-Wide Association Study of Salinity Tolerance During Germination in Barley (Hordeum vulgare L.)
Source: Front Plant Sci. 2020 Feb 21;11:118. doi: 10.3389/fpls.2020.00118 (PMC7047234; doi:10.3389/fpls.2020.00118)
Supplement: Supplementary file 9 [file Table_4.docx]

| **Supplementary Table 4:** The top 10 best and worst salinity-tolerant accessions and Australian commercial varieties based on the salinity tolerance index at two sites (Merredin and Katanning) and their average. | | | | |
| --- | --- | --- | --- | --- |
|  |  | **Salinity tolerance index** | | |
|  |  | **Merredin** | **Katanning** | **Average** |
| **Top 10 best accessions** | | | | |
| 1 | WABAR2347 | 98.98 | 95.00 | 96.99 |
| 2 | Har.Nan-35- | 100.00 | 90.00 | 96.09 |
| 3 | BM9647D-66 | 98.67 | 93.00 | 95.84 |
| 4 | 90SM193-34- | 97.92 | 91.92 | 94.92 |
| 5 | WVA22 | 94.77 | 95.00 | 94.88 |
| 6 | WABAR2234 | 100.00 | 87.00 | 94.59 |
| 7 | CDCGuardian | 94.77 | 94.00 | 94.38 |
| 8 | Yambla | 93.75 | 95.00 | 94.38 |
| 9 | 90S205-45-4 | 94.44 | 94.00 | 94.22 |
| 10 | H92036005Z | 95.79 | 91.89 | 93.84 |
|  | **Mean** | **96.91** | **92.68** | **95.01** |
| **Top 10 worst accessions** | | | | |
| 1 | HB09309 | 54.01 | 61.55 | 57.78 |
| 2 | CLE268 | 56.49 | 59.00 | 57.74 |
| 3 | C2-05-63/71 | 63.25 | 51.43 | 57.34 |
| 4 | ESPERANCEOR | 58.57 | 55.75 | 57.16 |
| 5 | EB1112 | 56.00 | 56.04 | 56.02 |
| 6 | 96B543 | 63.46 | 46.96 | 55.21 |
| 7 | 04S213D-B-1 | 55.04 | 54.97 | 55.01 |
| 8 | Torrens | 55.47 | 50.00 | 52.73 |
| 9 | Shepherd | 59.92 | 51.56 | 55.74 |
| 10 | Inari | 57.69 | 52.10 | 54.90 |
|  | **Mean** | **57.99** | **53.94** | **55.96** |
| **Representative of Australian commercial varieties** | | | | |
| 1 | Flinders | 80.44 | 78.43 | 79.43 |
| 2 | GrangeR | 73.62 | 75.00 | 74.31 |
| 3 | Buloke | 69.00 | 64.67 | 66.84 |
| 4 | Commander | 65.91 | 63.00 | 64.45 |
| 5 | Hamelin | 79.82 | 82.31 | 81.06 |
| 6 | Vlamingh | 76.32 | 81.63 | 78.98 |
| 7 | Compass | 84.63 | 65.63 | 75.13 |
| 8 | Lockyer | 86.27 | 68.18 | 77.23 |
|  | **Mean** | **77.00** | **72.36** | **74.68** |
