# Supplementary material for: Genome-Wide Association Study of Salinity Tolerance During Germination in Barley (Hordeum vulgare L.)
Source: Front Plant Sci. 2020 Feb 21;11:118. doi: 10.3389/fpls.2020.00118 (PMC7047234; doi:10.3389/fpls.2020.00118)
Supplement: Supplementary file 10 [file Table_5.docx]

| **Supplementary Table 5**: Genes in QTN flanking regions of significant markers associated with salinity tolerance during germination | | | | | |
| --- | --- | --- | --- | --- | --- |
| **Marker** | **Chro** | **Genes associated ID** | **Start** | **End** | **Function description** |
| L1H018492689 | 1H | HORVU1Hr1G008110.1 | 17447097 | 17449114 | B3 domain-containing protein |
|  | 1H | HORVU1Hr1G008120.1 | 17566211 | 17571908 | dehydroascorbate reductase 2 |
|  | 1H | HORVU1Hr1G008130.1 | 17580274 | 17583426 | 11S seed storage protein |
|  | 1H | HORVU1Hr1G008140.1 | 17584434 | 17585571 | TSA: Wollemia nobilis Ref_Wollemi_Transcript_13814 |
|  | 1H | HORVU1Hr1G008150.1 | 17623756 | 17625773 | B3 domain-containing protein |
|  | 1H | HORVU1Hr1G008160.3 | 17670835 | 17672723 | COP9 signalosome complex subunit 5b |
|  | 1H | HORVU1Hr1G008170.2 | 17712400 | 17712697 | Ubiquitin-like superfamily protein |
|  | 1H | HORVU1Hr1G008230.4 | 17901407 | 17905134 | dual specificity protein phosphatase 1 |
|  | 1H | HORVU1Hr1G008250.12 | 17907383 | 17913396 | beta-hexosaminidase 1 |
|  | 1H | HORVU1Hr1G008270.1 | 17919392 | 17919843 | Anthocyanin 5-aromatic acyltransferase |
|  | 1H | HORVU1Hr1G008290.1 | 18102516 | 18109705 | MADS-box transcription factor 27 |
|  | 1H | HORVU1Hr1G008300.3 | 18158350 | 18163296 | MADS-box transcription factor 27 |
|  | 1H | HORVU1Hr1G008330.5 | 18353095 | 18359860 | evolutionarily conserved C-terminal region 5 |
|  | 1H | HORVU1Hr1G008340.2 | 18398661 | 18399623 | unknown function |
|  | 1H | HORVU1Hr1G008350.2 | 18408388 | 18414274 | UDP-galactose transporter 5 |
|  | 1H | HORVU1Hr1G008360.2 | 18458166 | 18463957 | laccase 7 |
|  | 1H | HORVU1Hr1G008380.1 | 18468091 | 18468734 | Heparanase-like protein 2 |
|  | 1H | HORVU1Hr1G008370.2 | 18472177 | 18474612 | undescribed protein |
|  | 1H | HORVU1Hr1G008420.1 | 18484404 | 18485253 | Piriformospora indica-insensitive protein 2 |
|  | 1H | HORVU1Hr1G008430.1 | 18487438 | 18488152 | undescribed protein |
|  | 1H | HORVU1Hr1G008440.2 | 18489346 | 18490548 | unknown function |
| C1H556900757 | 1H | HORVU1Hr1G094980.1 | 556900386 | 556904840 | Early flowering 3 |
|  | 1H | HORVU1Hr1G094990.2 | 556905147 | 556910542 | Protein kinase superfamily protein |
|  | 1H | HORVU1Hr1G095010.1 | 556924891 | 556929224 | Tudor/PWWP/MBT superfamily protein |
|  | 1H | HORVU1Hr1G095020.1 | 556950985 | 556953259 | casein kinase I-like 5 |
|  | 1H | HORVU1Hr1G095060.2 | 557105950 | 557109459 | U6 snRNA-associated Sm-like protein LSm8 |
|  | 1H | HORVU1Hr1G095080.1 | 557108008 | 557115788 | WD-40 repeat family protein / notchless protein; p |
|  | 1H | HORVU1Hr1G095090.1 | 557118354 | 557118541 | U-box domain-containing protein 4 |
|  | 1H | HORVU1Hr1G095100.1 | 557122078 | 557125772 | ATP-dependent RNA helicase DeaD |
|  | 1H | HORVU1Hr1G095110.2 | 557387532 | 557391034 | unknown function |
|  | 1H | HORVU1Hr1G095120.1 | 557544024 | 557544124 | cDNA clone:001-043-A08; full insert sequence |
|  | 1H | HORVU1Hr1G095130.3 | 557707978 | 557709129 | unknown function |
|  | 1H | HORVU1Hr1G095140.1 | 557764284 | 557767320 | histone deacetylase 2B |
|  | 1H | HORVU1Hr1G095150.1 | 557774147 | 557779964 | AT1 protein |
|  | 1H | HORVU1Hr1G095160.1 | 557826700 | 557826926 | undescribed protein |
|  | 1H | HORVU1Hr1G095170.6 | 557837872 | 557840541 | Rho GDP-dissociation inhibitor 1 |
|  | 1H | HORVU1Hr1G095180.1 | 557898136 | 557899011 | unknown function |
|  | 1H | HORVU1Hr1G095190.1 | 557908922 | 557909609 | undescribed protein |
|  | 1H | HORVU1Hr1G095210.4 | 557940300 | 557942187 | unknown function |
|  | 1H | HORVU1Hr1G095220.1 | 557944582 | 557946336 | RING/U-box superfamily protein |
|  | 1H | HORVU1Hr1G095230.35 | 557948038 | 557952685 | structural maintenance of chromosomes 5 |
|  | 1H | HORVU1Hr1G095240.1 | 557960891 | 557964049 | Two-component response regulator ORR42 |
|  | 1H | HORVU1Hr1G095250.1 | 557981437 | 557982203 | unknown function |
|  | 1H | HORVU1Hr1G095270.16 | 557990565 | 557997508 | Zinc finger CCCH domain-containing protein 37 |
|  | 1H | HORVU1Hr1G095300.15 | 558019323 | 558024901 | Argonaute family protein |
|  | 1H | HORVU1Hr1G095310.7 | 558025868 | 558026702 | Reticulon family protein |
|  | 1H | HORVU1Hr1G095330.1 | 558119435 | 558121492 | Eukaryotic aspartyl protease family protein |
|  | 1H | HORVU1Hr1G095340.3 | 558135613 | 558140825 | Leucine-rich receptor-like protein kinase family p |
|  | 1H | HORVU1Hr1G095370.1 | 558185054 | 558186084 | undescribed protein |
|  | 1H | HORVU1Hr1G095390.1 | 558216056 | 558217013 | Zinc finger protein CONSTANS-LIKE 4 |
|  | 1H | HORVU1Hr1G095400.1 | 558218311 | 558221262 | Pleckstrin homology (PH) domain-containing protein |
| L7H212035410 | 7H | HORVU7Hr1G053930.9 | 212741878 | 212744393 | lipase 1 |
|  | 7H | HORVU7Hr1G053940.1 | 213013811 | 213019550 | uridine kinase-like 2 |
|  | 7H | HORVU7Hr1G053950.1 | 213078230 | 213078879 | Late embryogenesis abundant (LEA) hydroxyproline-r |
|  | 7H | HORVU7Hr1G053970.1 | 213255282 | 213255655 | 1-aminocyclopropane-1-carboxylate synthase 11 |
|  | 7H | HORVU7Hr1G054000.4 | 213613194 | 213616315 | Chromosome 3B; genomic scaffold; cultivar Chinese |
|  | 7H | HORVU7Hr1G054010.1 | 213651429 | 213654111 | 60S ribosomal protein L17-2 |
|  | 7H | HORVU7Hr1G054020.2 | 213855291 | 213857668 | exocyst subunit exo70 family protein F1 |
|  | 7H | HORVU7Hr1G054040.1 | 213889904 | 213890312 | Major facilitator superfamily protein |
|  | 7H | HORVU7Hr1G054060.3 | 214453499 | 214459125 | Nitrate reductase [NADH] |
|  | 7H | HORVU7Hr1G054070.1 | 214455835 | 214461060 | Far1-related sequence 3 isoform 1 |
|  | 7H | HORVU7Hr1G054090.4 | 214811865 | 214815839 | purple acid phosphatase 27 |
|  | 7H | HORVU7Hr1G054120.1 | 215037587 | 215037757 | Chlorophyll synthase; chloroplastic |
|  | 7H | HORVU7Hr1G054130.4 | 215040274 | 215045086 | microtubule-associated protein 65-8 |
|  | 7H | HORVU7Hr1G054140.1 | 215040171 | 215062445 | Transducin family protein / WD-40 repeat family pr |
|  | 7H | HORVU7Hr1G054160.3 | 215063343 | 215070088 | Serine/threonine-protein kinase Rio1 |
|  | 7H | HORVU7Hr1G054190.14 | 215393914 | 215397113 | Threonylcarbamoyl-AMP synthase |
|  | 7H | HORVU7Hr1G054220.1 | 216028773 | 216035915 | MADS-box transcription factor 7 |
|  | 7H | HORVU7Hr1G054230.1 | 216113703 | 216120774 | Protein kinase superfamily protein |
|  | 7H | HORVU7Hr1G054320.1 | 216662723 | 216664112 | MADS-box transcription factor family protein |
|  | 7H | HORVU7Hr1G054360.1 | 216704870 | 216706683 | glutamate-1-semialdehyde-2;1-aminomutase |
|  | 7H | HORVU7Hr1G054380.1 | 216784253 | 216786999 | glutamate-1-semialdehyde-2;1-aminomutase |
|  | 7H | HORVU7Hr1G054390.1 | 216788559 | 216822414 | MADS-box transcription factor family protein |
|  | 7H | HORVU7Hr1G054440.1 | 217284728 | 217287615 | nuclear transport factor 2A |
|  | 7H | HORVU7Hr1G054460.2 | 217289596 | 217289820 | NAM-like protein |
|  | 7H | HORVU7Hr1G054500.2 | 217854577 | 217858795 | Nodulin-like / Major Facilitator Superfamily prote |
|  | 7H | HORVU7Hr1G054510.1 | 218220114 | 218223625 | Peroxidase superfamily protein |
|  | 7H | HORVU7Hr1G054520.2 | 218228667 | 218230555 | zinc ion binding;transcription regulators |
|  | 7H | HORVU7Hr1G054530.1 | 218520818 | 218524923 | Lipid transfer protein-like |
|  | 7H | HORVU7Hr1G054550.1 | 218865361 | 218869367 | Transmembrane emp24 domain-containing protein |
|  | 7H | HORVU7Hr1G054580.1 | 219675948 | 219676121 | Tubby-like F-box protein 9 |
|  | 7H | HORVU7Hr1G054610.3 | 220370264 | 220373270 | Bifunctional uridylyltransferase/uridylyl-removing |
|  | 7H | HORVU7Hr1G054660.6 | 221197966 | 221199221 | Chromosome 3B; genomic scaffold; cultivar Chinese |
|  | 7H | HORVU7Hr1G054670.1 | 221347389 | 221350163 | 60S ribosomal protein L7a |
|  | 7H | HORVU7Hr1G054690.4 | 221603430 | 221606339 | ARM repeat superfamily protein |
|  | 7H | HORVU7Hr1G054710.2 | 221745516 | 221747264 | Sugar transporter SWEET |
|  | 7H | HORVU7Hr1G054730.9 | 221809550 | 221814569 | Membrane fusion protein Use1 |
|  | 7H | HORVU7Hr1G054760.2 | 221851240 | 221855098 | Protein S-acyltransferase 8 |
|  | 7H | HORVU7Hr1G054850.2 | 222085959 | 222089913 | Multiple organellar RNA editing factor 3; mitochon |
|  | 7H | HORVU7Hr1G054880.1 | 222639811 | 222641222 | basic helix-loop-helix (bHLH) DNA-binding superfam |
|  | 7H | HORVU7Hr1G054890.2 | 222644027 | 222646598 | B12D protein |
|  | 7H | HORVU7Hr1G054910.1 | 222704979 | 222706366 | Leucine-rich repeat protein kinase family protein |
|  | 7H | HORVU7Hr1G054920.1 | 222709151 | 222711551 | Mitochondrial import inner membrane translocase su |
|  | 7H | HORVU7Hr1G054980.28 | 222924348 | 222932515 | Glycerophosphodiester phosphodiesterase GDE1 |
|  | 7H | HORVU7Hr1G055010.1 | 222934962 | 222936149 | RING/U-box superfamily protein |
|  | 7H | HORVU7Hr1G055080.2 | 223284188 | 223288321 | NAC domain protein; |
|  | 7H | HORVU7Hr1G055090.3 | 223540999 | 223548166 | Pyruvate dehydrogenase E1 component subunit beta |
|  | 7H | HORVU7Hr1G055100.6 | 223549926 | 223554903 | unknown function |
|  | 7H | HORVU7Hr1G055160.1 | 225222379 | 225222765 | unknown function |
|  | 7H | HORVU7Hr1G055180.1 | 225250259 | 225253008 | Transcription factor bHLH137 |
|  | 7H | HORVU7Hr1G055190.1 | 225320897 | 225321325 | trehalose-6-phosphate synthase |
|  | 7H | HORVU7Hr1G055200.1 | 225322578 | 225323273 | Elongated mesocotyl1 |
|  | 7H | HORVU7Hr1G055220.4 | 225589997 | 225616772 | ADP-ribosylation factor GTPase-activating protein |
|  | 7H | HORVU7Hr1G055270.2 | 225748834 | 225749980 | Histone-lysine N-methyltransferase |
|  | 7H | HORVU7Hr1G055280.1 | 225947017 | 225950048 | Ubiquitin thioesterase otubain-like |
|  | 7H | HORVU7Hr1G055290.6 | 225951783 | 225965924 | O-fucosyltransferase family protein |
|  | 7H | HORVU7Hr1G055300.1 | 225960676 | 225960924 | undescribed protein |
|  | 7H | HORVU7Hr1G055310.1 | 226129155 | 226130687 | Cathepsin B-like cysteine proteinase 6 |
|  | 7H | HORVU7Hr1G055320.1 | 226172292 | 226173072 | Heat stress transcription factor C-2a |
|  | 7H | HORVU7Hr1G055330.1 | 226285016 | 226286367 | rRNA N-glycosidase |
|  | 7H | HORVU7Hr1G055340.1 | 226514183 | 226519506 | Glycine--tRNA ligase |
|  | 7H | HORVU7Hr1G055370.6 | 226851895 | 226866971 | receptor-like protein kinase 1 |
|  | 7H | HORVU7Hr1G055390.1 | 226892424 | 226893019 | chitin elicitor receptor kinase 1 |
| D7H085710245 | 7H | HORVU7Hr1G036090.2 | 82420627 | 82440422 | kinesin 4 |
|  | 7H | HORVU7Hr1G036100.6 | 82606397 | 82608783 | laccase 14 |
|  | 7H | HORVU7Hr1G036110.1 | 82650592 | 82654618 | Mediator of RNA polymerase II transcription subuni |
|  | 7H | HORVU7Hr1G036120.1 | 82861778 | 82862187 | Speckle-type POZ protein-like |
|  | 7H | HORVU7Hr1G036130.1 | 82946101 | 82953029 | MADS-box transcription factor 55 |
|  | 7H | HORVU7Hr1G036140.1 | 82948556 | 82952709 | Elongation factor Ts |
|  | 7H | HORVU7Hr1G036160.1 | 83249502 | 83255889 | Peptidyl-prolyl cis-trans isomerase D |
|  | 7H | HORVU7Hr1G036180.1 | 83278444 | 83279568 | Copper ion-binding protein; putative |
|  | 7H | HORVU7Hr1G036170.8 | 83280307 | 83285878 | Pentatricopeptide repeat-containing protein |
|  | 7H | HORVU7Hr1G036210.1 | 83526144 | 83527023 | Cullin-associated NEDD8-dissociated protein 1 |
|  | 7H | HORVU7Hr1G036220.2 | 83526145 | 83528738 | Leucine-rich repeat protein kinase family protein |
|  | 7H | HORVU7Hr1G036280.1 | 83610089 | 83611469 | Protein CURVATURE THYLAKOID 1D; chloroplastic |
|  | 7H | HORVU7Hr1G036290.1 | 83636776 | 83636944 | undescribed protein |
|  | 7H | HORVU7Hr1G036310.3 | 83687930 | 83688669 | alpha/beta-Hydrolases superfamily protein |
|  | 7H | HORVU7Hr1G036330.1 | 84006342 | 84008301 | Signal recognition particle 54 kDa protein 2 |
|  | 7H | HORVU7Hr1G036370.1 | 84228373 | 84229694 | RING/U-box superfamily protein |
|  | 7H | HORVU7Hr1G036380.2 | 84258258 | 84259714 | RING/U-box superfamily protein |
|  | 7H | HORVU7Hr1G036390.1 | 84513601 | 84514975 | Blue copper protein |
|  | 7H | HORVU7Hr1G036410.1 | 84565373 | 84565873 | unknown function |
|  | 7H | HORVU7Hr1G036420.10 | 84566492 | 84569524 | LMBR1 domain-containing protein 2 homolog B |
|  | 7H | HORVU7Hr1G036430.2 | 84582203 | 84583778 | DNA helicase MCM9 |
|  | 7H | HORVU7Hr1G036440.1 | 84627463 | 84627646 | DNA replication licensing factor MCM9 |
|  | 7H | HORVU7Hr1G036450.20 | 84642784 | 84646994 | O-fucosyltransferase family protein |
|  | 7H | HORVU7Hr1G036460.1 | 84700862 | 84702674 | PATATIN-like protein 4 |
|  | 7H | HORVU7Hr1G036470.3 | 84846602 | 85031958 | heat shock protein 21 |
|  | 7H | HORVU7Hr1G036500.7 | 84965457 | 84973258 | heat shock protein 21 |
|  | 7H | HORVU7Hr1G036520.4 | 85198618 | 85200204 | Pectin lyase-like superfamily protein |
|  | 7H | HORVU7Hr1G036540.3 | 85341216 | 85462034 | heat shock protein 21 |
|  | 7H | HORVU7Hr1G036560.1 | 85490557 | 85490959 | Chitinase family protein |
|  | 7H | HORVU7Hr1G036570.2 | 85583651 | 85584754 | heat shock protein 21 |
