# Supplementary material for: Genome-Wide Association Study of Salinity Tolerance During Germination in Barley (Hordeum vulgare L.)
Source: Front Plant Sci. 2020 Feb 21;11:118. doi: 10.3389/fpls.2020.00118 (PMC7047234; doi:10.3389/fpls.2020.00118)
Supplement: Supplementary file 11 [file Table_6.docx]

| **Supplementary Table 6**: Multiple comparisons of subpopulations mean tolerance index | | | | | |
| --- | --- | --- | --- | --- | --- |
| **Subpopulation** | **T. Index** | **Comparison** | **Mean Difference** | **Std. Error** | **Sig.** |
| 1 | 79.43 | - | - | - | - |
|  |  | 2 | 2.13 | 2.78 | 0.44 |
|  |  | 3 | 0.06 | 2.62 | 0.98 |
|  |  | 4 | 0.47 | 1.84 | 0.80 |
|  |  | 5 | 2.15 | 2.12 | 0.31 |
|  |  | 6 | 4.78 | 2.52 | 0.06 |
| 2 | 77.30 | 3 | -2.07 | 2.94 | 0.48 |
|  |  | 4 | -1.66 | 2.28 | 0.47 |
|  |  | 5 | 0.03 | 2.51 | 0.99 |
|  |  | 6 | 2.66 | 2.86 | 0.35 |
| 3 | 79.37 | 4 | 0.41 | 2.08 | 0.84 |
|  |  | 5 | 2.10 | 2.33 | 0.37 |
|  |  | 6 | 4.73 | 2.70 | 0.08 |
| 4 | 78.96 | 5 | 1.69 | 1.40 | 0.23 |
|  |  | 6 | 4.32* | 1.96 | 0.03 |
| 5 | 77.27 | 6 | 2.63 | 2.23 | 0.24 |
| 6 | 74.64 | - | - | - | - |
|  | * The mean difference is significant at the 0.05 level. | | | | |
